# Mutations in or near the Transmembrane Domain Alter PMEL Amyloid Formation from Functional to Pathogenic

**DOI:** 10.1371/journal.pgen.1002286

**Published:** 2011-09-15

**Authors:** Brenda Watt, Danièle Tenza, Mark A. Lemmon, Susanne Kerje, Graça Raposo, Leif Andersson, Michael S. Marks

**Affiliations:** 1 Department of Pathology and Laboratory Medicine and Department of Physiology, University of Pennsylvania, Philadelphia, Pennsylvania, United States of America; 2 Cell and Molecular Biology Graduate Group, University of Pennsylvania, Philadelphia, Pennsylvania, United States of America; 3 Institut Curie, Centre de Recherche, Paris, France; 4 CNRS, UMR-144, Paris, France; 5 Department of Biochemistry and Biophysics, University of Pennsylvania, Philadelphia, Pennsylvania, United States of America; 6 Science for Life Laboratory, Department of Medical Biochemistry and Microbiology, Uppsala University, Uppsala, Sweden; 7 Department of Animal Breeding and Genetics, Swedish University of Agricultural Sciences, Uppsala, Sweden; Medical Research Council Human Genetics Unit, United Kingdom

## Abstract

PMEL is a pigment cell-specific protein that forms physiological amyloid fibrils upon which melanins ultimately deposit in the lumen of the pigment organelle, the melanosome. Whereas hypomorphic PMEL mutations in several species result in a mild pigment dilution that is inherited in a recessive manner, *PMEL* alleles found in the *Dominant white* (*DW*) chicken and *Silver* horse (*HoSi*)—which bear mutations that alter the PMEL transmembrane domain (TMD) and that are thus outside the amyloid core—are associated with a striking loss of pigmentation that is inherited in a dominant fashion. Here we show that the *DW* and *HoSi* mutations alter PMEL TMD oligomerization and/or association with membranes, with consequent formation of aberrantly packed fibrils. The aberrant fibrils are associated with a loss of pigmentation in cultured melanocytes, suggesting that they inhibit melanin production and/or melanosome integrity. A secondary mutation in the *Smoky* chicken, which reverts the dominant *DW* phenotype, prevents the accumulation of PMEL in fibrillogenic compartments and thus averts DW–associated pigment loss; a secondary mutation found in the *Dun* chicken likely dampens a *HoSi*–like dominant mutation in a similar manner. We propose that the *DW* and *HoSi* mutations alter the normally benign amyloid to a pathogenic form that antagonizes melanosome function, and that the secondary mutations found in the *Smoky* and *Dun* chickens revert or dampen pathogenicity by functioning as null alleles, thus preventing the formation of aberrant fibrils. We speculate that PMEL mutations can model the conversion between physiological and pathological amyloid.

## Author Summary

Amyloid is a protein fold that is normally associated with pathology, such as neurodegeneration in Alzheimer, Parkinson, and Creutzfeldt–Jakob diseases. The amyloid fold has also been exploited by nature for functional purposes; for example, proteolytic fragments of the pigment cell-specific integral membrane protein, PMEL, form amyloid fibrils upon which melanin pigments polymerize within subcellular organelles called melanosomes. Whereas animal models that entirely lack PMEL expression have modest pigment loss, chickens or horses with small in-frame mutations that alter a non-amyloidogenic region of PMEL have severe pigment loss that is thought to be associated with pigment cell death. We show here that these mutations alter the capacity of this region to self-associate, likely changing the conformation of full-length PMEL oligomers. While these changes do not affect the intrinsic ability of PMEL to form amyloid fibrils, they alter either the fibrils themselves or the kinetics of fibril formation such that they form more compact structures and inhibit melanin formation when expressed in melanocytes in culture. Based on our results, we speculate that minor alterations in pre-amyloid assembly of an amyloidogenic protein influences entry into either a benign or a pathogenic amyloid pathway.

## Introduction

Amyloid fibrils are polymers of single proteins that oligomerize and assemble into a characteristic fibrillar structure with a cross-beta sheet backbone [1], [2]. Amyloid formation is typically associated with pathologies, such as the Aβ aggregates in Alzheimer Disease or prion aggregates in inherited or acquired spongiform encephalopathies. However, the amyloid fold has also been exploited for functional means in prokaryotes, lower eukaryotes and even in mammals [2]–[6]. The structural and biogenetic features that distinguish functional from pathological amyloid are not well understood. Discerning these features might lead to novel therapies for amyloid diseases.

**Figure 1:**
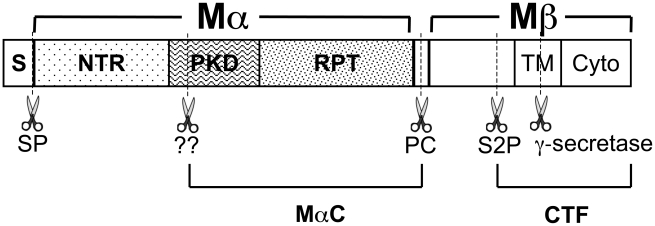
Schematic diagram of PMEL proteolytic processing. Diagram of PMEL domain structure, relevant proteolytic fragments and proteolytic cleavage sites (dashed lines and scissors). Within the primary sequence are indicated the signal peptide (S), N-terminal region (NTR), polycystic kidney disease repeat domain (PKD), repeat domain (RPT), transmembrane domain (TM) and cytoplasmic domain (Cyto). The signal peptide is removed from the N-terminus of PMEL in the ER by signal peptidase (SP). PMEL is cleaved by a proprotein convertase (PC) to produce Mα and Mβ fragments, which are linked by disulfide bonds (not shown). Subsequent cleavage by a site 2 protease (S2P) at a site proximal to the lumenal side of the TM produces a C-terminal fragment (CTF) and liberates Mα. As yet unidentified enzymes (marked “??”) further cleave Mα at as yet unknown sites to produce MαC. MαC fragments and a fragment of the PKD domain have been shown to accumulate in fibrillar stage II melanosomes [23].

A potential model for distinguishing functional from pathological amyloids is the pigment cell-specific integral membrane glycoprotein, PMEL (also called gp100, Pmel17, or Silver) [7], [8]. Functional amyloid fibrillar sheets, composed largely of lumenal proteolytic fragments of PMEL, form the structural foundation of eumelanosomes, which are membrane-bound, pigment cell-specific lysosome-related organelles within which black and brown melanin pigments are synthesized and stored [9], [10]. The fibrils begin to form in association with intralumenal membrane vesicles (ILVs) within multivesicular melanosome precursors [11], [12], to which PMEL is selectively delivered during biosynthetic transport [13], [14]. Either in the trans Golgi network [15] or in association with the ILVs or with membrane domains destined for the ILVs [16], PMEL is cleaved by two site-specific proteases to liberate a lumenal fragment called Mα [13], [14], [16]–[18] (see Figure 1). Mα then undergoes ordered oligomerization into protofibrils that are detergent insoluble [17], detectable by electron microscopy (EM) [11], [13], [14], [17], and reactive with amyloidogenic dyes [19]. The Mα fibrils are further matured by proteolytic processing [20]–[23] and assemble into sheets [11], [24], upon which melanins deposit as they are synthesized during melanosome maturation [10], [24]. In vitro, denatured recombinant Mα fragments that are diluted into non-denaturing buffers rapidly assemble into fibrils that are classified as amyloid by a number of biophysical measures [19], [23], [25]. The physiological function of the fibrils is not entirely clear, but they likely serve to condense melanin intermediates to facilitate their detoxification, polymerization, and/or intercellular or intracellular transfer [8]. This function seems to be important for optimal pigmentation, as animal models with apparently hypomorphic mutations in PMEL show varying levels of hypopigmentation, ranging from modest in the *silver* mouse [26] to more pronounced in *Merle* dogs [27] and *fading vision* zebrafish [28]. In the accompanying paper by Hellström *et al*. [29], we show that a complete loss of PMEL expression in the *Pmel ^-/-^* mouse also presents with a modest pigment dilution.

Compared to the more modest effects of the PMEL mutations described above, genetic models in the chicken and horse show that in some cases, PMEL mutations can result in severe hypopigmentation. In the chicken, a nine bp in-frame insertion within the coding region for the PMEL transmembrane domain (TMD; TM^insWAP^) is associated with the nearly complete loss of feather eumelanin in *Dominant White* (*DW*) chickens [30]. The pigment loss in *DW* chickens is associated with poor melanocyte survival in culture and with melanocyte depletion along the feather shaft in vivo [31], both exaggerations of the increased cell cycle length [32] and slow, progressive melanocyte loss [33] observed in homozygous mice bearing the recessive, hypomorphic PMEL *silver* mutation. However, unlike mouse *silver*, the *DW* allele is associated with impaired melanosome maturation and with melanosome loss within epidermal melanocytes [34], [35]. Moreover, the *DW* allele differs from mouse *silver* and other PMEL hypomorphs in that it confers hypopigmentation in a dominant fashion, suggesting that the *DW* PMEL product must either inhibit endogenous PMEL function in a dominant-negative manner or confer some gain-of-function that is detrimental to melanocyte function and/or health. Interestingly, a concomitant deletion within the conserved lumenal Polycystic Kidney Disease-1 homology (PKD) domain in the *Smoky* chicken (PKD^ΔLVVT^) reverts the dominant phenotype of the TM^insWAP^ *DW* mutation, resulting in a much more modest hypopigmentation that is inherited in a recessive manner [30]. In *Silver* horses (*HoSi*), a dominant missense mutation in PMEL results in a transversion that substitutes a cysteine for the second of three consecutive arginine residues immediately following the PMEL TMD (TM^R625C^), causing a dilution of black pigment that is most noticeable in the mane and tail of the animal [36], [37]. An orthologously identical TM^R625C^ mutation in the *Dun* chicken *PMEL* allele is accompanied by an additional gene deletion (TM^Δ5^) that eliminates five residues from the PMEL TMD [30]. However, while *Dun* is likewise dominant, it confers a more modest hypopigmentation, suggesting either species-specific variation in the TM^R625C^ PMEL phenotype or that the TM^Δ5^ secondary mutation might partially dampen the pigmentation defect that the TM^R625C^ mutation confers individually. Why the mutations in the *DW* chicken and *Silver* horse present a dominant phenotype and how they might be reverted by the additional deletions in the *Smoky* and (perhaps) *Dun* chickens is not understood.

Given the severity of their pigmentation phenotype, the association with loss of melanosome integrity and melanocyte viability *in vivo* and in cell culture, and the dominant nature of the mutations, we hypothesized that the TM^insWAP^ and TM^R625C^ mutations of PMEL in the *DW* chicken and the *Silver* horse, respectively, alter the cellular or biophysical properties of PMEL to ultimately convert functional amyloid into a pathological form. Here, by recapitulating these mutations in the context of human PMEL (hPMEL), we provide evidence to support this hypothesis. Moreover, we show that the secondary PKD^ΔLVVT^ and TM^Δ5^ mutations in the *Smoky* and *Dun* chicken PMEL orthologues represent null or partial null alleles that revert the pathological effects of the TM^insWAP^ and TM^R625C^ mutations on pigmentation. Finally, we show that changes in or near the TMD of an integral membrane amyloidogenic protein can influence the oligomerization of a distal lumenal fragment into functional amyloid. We discuss these findings with regard to their potential implications for the formation of functional *vs.* pathological amyloid.

## Results

### Mutations found in *DW* and *HoSi* PMEL alter the oligomerization potential of the human PMEL transmembrane domain

**Figure 2:**
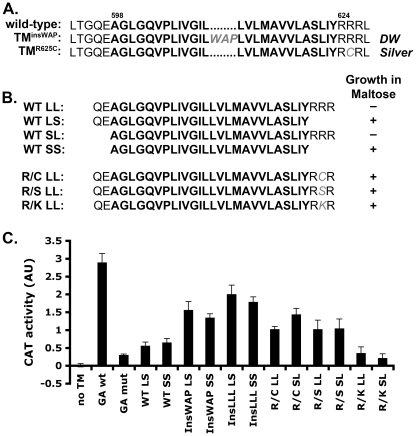
PMEL TMD mutations cause increased TMD–mediated dimerization. A. Wild-type human PMEL (hPMEL) TMD sequence (bold) and surrounding amino acids. In the context of hPMEL, homologous mutations to those found in the *DW* chicken (TM^insWAP^) and *Silver* horse (TM^R625C^) are shown in gray. B. *Left*, TMD sequences (bold) used to make the ToxR-TMD-MBP chimeras with (L) or without (S) the N-terminal boundary residues QE and C-terminal boundary residues RRR. *Right*, MBP-deficient bacteria transformed with the indicated TMD-containing chimera were plated on medium with maltose as the only source of carbon; shown is whether colonies grew (+) or not (-). Growth indicates proper insertion of the chimera into the bacterial membrane. C. Representative histogram (from at least 3 similar experiments) of CAT activity, representing TMD-mediated dimerization of the chimera, as measured spectrophotometrically and normalized to protein concentration. GA, Glycophorin A TM domain. Bars, standard deviation.

Human PMEL (hPMEL) forms pre-amyloid oligomers that are stabilized by disulfide bonds [13], and a recent study found that hPMEL formed fewer disulfide-bonded oligomers when coexpressed with chicken PMEL bearing the *DW*-associated TM^insWAP^ mutation [38]. However, it is not known whether the result in that study reflected a direct effect of the TM^insWAP^ mutation or an inability of hPMEL to form the appropriate disulfide bonds with chicken PMEL. To specifically test whether and how mutations in or near the PMEL TMD influence PMEL oligomerization, we introduced mutations analogous to those found in the *DW* chicken (TM^insWAP^) and *Silver* horse (TM^R625C^) in the context of hPMEL (Figure 2A). Upon expression of these mutants or wild-type full-length hPMEL in HeLa cells, we found that the TMD mutants were equally effective as wild-type hPMEL at forming disulfide bonded oligomers, as detected by non-reducing SDS-PAGE and immunoblotting (Figure S1A). These data suggest that the PMEL mutations associated with the *DW* chicken and *HoSi* do not affect the formation of disulfide-bonded PMEL oligomers when presented within the context of hPMEL. The previously observed reduction in disulfide-bonded oligomers by hPMEL upon coexpression of chicken *DW* PMEL [38] thus likely reflects the inability of these cross-species PMEL isoforms to form oligomers, which is supported by our inability to co-immunoprecipitate wild-type mouse PMEL with wild-type hPMEL (data not shown).

While oligomerization of the hPMEL lumenal domain is stabilized by disulfide bonds, it is not known whether the TMD itself has oligomeric properties that might otherwise influence lumenal domain interactions. To determine whether the hPMEL TMD can oligomerize on its own and whether the TM^insWAP^ and TM^R625C^ mutations influence this property, we turned to the widely used TOXCAT assay [39]. In this assay, a chimeric protein consisting of the transcription factor ToxR, the TMD of interest, and maltose binding protein (MBP) is expressed in MBP-deficient bacteria. Proper insertion of the chimeric protein into the plasma membrane confers growth in maltose as the only carbon source, and oligomerization mediated by the TMD activates ToxR and stimulates ToxR-dependent transcription of the chloramphenicol acetyl transferase (CAT) gene. Upon expression of ToxR-TMD-MBP chimeras containing the PMEL TMD with or without the natural border residues at either end (Figure 2B), only chimeras lacking the C-terminal border residues, RRR (SS, LS), conferred growth in maltose — regardless of the presence of the N-terminal border residues, QE — despite equivalent expression of all chimeras as determined by immunoblotting (Figure S1B). This suggested that the C-terminal residues interfered with insertion of the chimera into the plasma membrane with the proper orientation. Interestingly, altering the second arginine in the C-terminal border sequence to either Cys (R/C; as in the TM^R625C^ mutant), Ser (R/S), or Lys (R/K) allowed the chimeric protein to insert properly into the membrane, as evidenced by maltose complementation (Figure 2B). This indicates that the TM^R625C^ substitution associated with *HoSi* PMEL alters the properties of the PMEL TMD.

For those chimeras that confer growth in maltose, we then tested their ability to dimerize by measuring CAT activity using a spectrophotometric assay (Figure 2C) [40]. As a positive control we used a chimera containing the strongly dimerizing TMD of Glycophorin A (GA wt), and as a negative control we used either the vector without a TMD (no TM) or a chimera containing the non-dimerizing Glycophorin A G83I mutant TMD (GA mut) [39], [41]. The wild-type PMEL TMD, lacking (SS) or containing (LS) the N-terminal border residues, conferred similar CAT activity as the negative controls. In contrast, the TM^insWAP^ (InsWAP) and TM^R625C^ (R/C) mutations conferred substantial CAT activity. These data indicate that whereas the wild-type hPMEL TMD is not capable of oligomerization, both the *DW*-associated TMD insertion and the *HoSi*-associated R/C transversion facilitate TMD dimerization. Insertion of three leucines (InsLLL) in place of the WAP insertion conferred an even slightly higher CAT activity, suggesting that the increased dimerization mediated by the TM^insWAP^ mutation is likely due to the increase in TMD length. To test whether the R/C mutation reflected a specific property of the cysteine (such as disulfide bond formation), we tested whether replacement of the same arginine residue by serine (R/S) or lysine (R/K) affected CAT activity. Interestingly, whereas both of these mutants conferred proper insertion into the plasma membrane as indicated by growth in maltose, the R/S mutant, but not R/K, conferred CAT activity similar to that by the R/C mutant. This suggests that the dimerization conferred by the R/C mutant reflects decreased repulsion between adjacent basic RRR (or RKR) motifs.

### *DW*– and *HoSi*–associated TMD mutations do not affect PMEL trafficking to premelanosomal compartments or proteolytic activation

**Figure 3:**
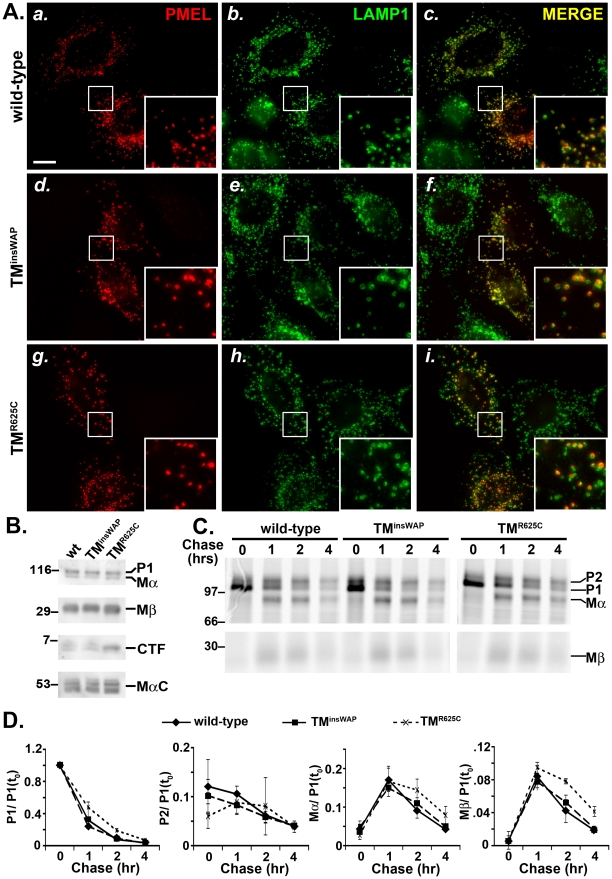
TMD mutations do not affect PMEL trafficking, maturation, or processing. HeLa cells were transiently transfected with low (A and C–D) or high (B) DNA levels of pCI plasmids encoding wild-type, TM^insWAP^ or TM^R625C^ hPMEL, and processed for analysis 48 hrs post-transfection. A. IFM analysis. HeLa cells were fixed and co-stained with antibodies against hPMEL (red; panels *a*, *d* and *g*) and LAMP1 (green; panels *b*, *e*, and *h*). Right panels (*c*, *f*, *i*) show the merged images, and insets show a 4X magnification of the boxed regions. Notice the similar pattern of labeling of wild-type (*a–c*), TM^insWAP^ (*d–f*) and TM^R625C^ hPMEL within LAMP1-labelled late endosomes/lysosomes. Bar, 20 µm. B. Immunoblot analysis. Transfected HeLa cells were lysed and fractionated into detergent soluble and insoluble fractions. Detergent soluble fractions (top three panels) were probed with antibodies to either the hPMEL N-terminus to detect P1 and Mα (top), or the C-terminus to detect Mβ and CTF (middle panels). Detergent insoluble, fibril-enriched fractions were probed with HMB45 to detect the PMEL-derived MαC fragments (lower panel). Left, molecular weight markers; right, relevant bands are indicated. C. Metabolic pulse chase/ immunoprecipitation analysis. Transfected HeLa cells were metabolically labeled with ^35^S methionine/ cysteine, chased for the indicated times, and lysed in Triton X-100. PMEL was immunoprecipitated from detergent soluble fractions using an antibody to the C-terminus. Left, molecular weight markers; right, relevant bands are indicated. D. Quantification of abundance of relevant PMEL fragments from at least three pulse chase experiments as shown in C.; the band intensity of each fragment was normalized to that of P1 at time zero [P1(t_0_)]. Bars, standard error.

We next tested whether the altered TMD properties associated with the TM^insWAP^ and TM^R625C^ mutations influenced PMEL trafficking or processing. hPMEL without (wild-type) or with the TM^insWAP^ or TM^R625C^ mutations was expressed ectopically in non-melanocytic HeLa cells by transient transfection. As previously shown, wild-type hPMEL expressed in these cells is enriched at steady state within late endosomes and lysosomes [13] (Figure 3Aa–c). Neither the TM^insWAP^ nor the TM^R625C^ mutation affected this steady state localization, as shown by the predominant labeling for these mutants by immunofluorescence microscopy (IFM) on the interior of structures labeled by the late endosome/lysosome marker, LAMP1 (Figure 3Ad–i). These data suggest that the TMD mutations in dominant PMEL mutants do not affect hPMEL trafficking, supporting a similar earlier conclusion for chicken *DW* PMEL [38].

In order to liberate the amyloidogenic Mα fragment, PMEL undergoes regulated proteolytic cleavage in the lumenal domain by a proprotein convertase (PC) and an as yet unidentified site 2 protease (S2P) [17], [18] (Figure 1). Mα is then further processed by as yet unidentified proteases during fibril maturation [12], [21]–[23]. The products of these cleavages are detected and semi-quantified by immunoblot analysis of detergent-soluble lysates from HeLa cells transfected with wild-type hPMEL; the characteristic Mα and Mβ fragments result from PC cleavage of full-length hPMEL [17], the C-terminal fragment (CTF) results from S2P cleavage of Mβ [18], and the MαC fragments that are enriched in detergent-insoluble fibrils result from further proteolysis of Mα [21] (Figure 1). Similar levels of all fragments are present in lysates from cells expressing TM^insWAP^ or TM^R625C^ hPMEL, with the exception of a slight increase in the levels of the CTF relative to Mβ in cells expressing the TM^R625C^ mutant (Figure 3B). Moreover, whereas wild-type and TM^insWAP^ hPMEL often form two species of CTF (see doublet in Figure 3B), TM^R625C^ hPMEL forms predominantly a single species. These data suggest that TM^insWAP^ and TM^R625C^ hPMEL are effectively cleaved to fibrillogenic fragments by the PC and by proteases within late endosomal compartments but that S2P cleavage is favored at one of two sites in TM^R625C^ hPMEL and the ensuing CTF is likely more stable than the wild-type CTF.

To test whether processing kinetics are altered by the TMD mutations, we analyzed PMEL maturation in transfected HeLa cells by metabolic pulse/ chase analysis of wild-type, TM^insWAP^, or TM^R625C^ hPMEL immunoprecipitated from detergent-soluble lysates. As shown in Figure 3C, both the TM^insWAP^ and TM^R625C^ mutant hPMEL were matured to the Golgi-processed P2 form, cleaved to the Mα/ Mβ forms, and disappeared from detergent-soluble lysates with roughly wild-type kinetics (Figure 3D). Moreover, for all hPMEL variants, Mα was secreted into the medium with similar kinetics and efficiency, and CTF was generated with similar kinetics (data not shown).

Altogether, these results indicate that the TMD mutations found in the *DW* chicken and the *Silver* horse affect neither the delivery of PMEL to late endocytic compartments nor its ability to be processed to amyloidogenic Mα and MαC fragments.

### hPMEL with TM^insWAP^ and TM^R625C^ mutations is fibrillogenic within endosomes

**Figure 4:**
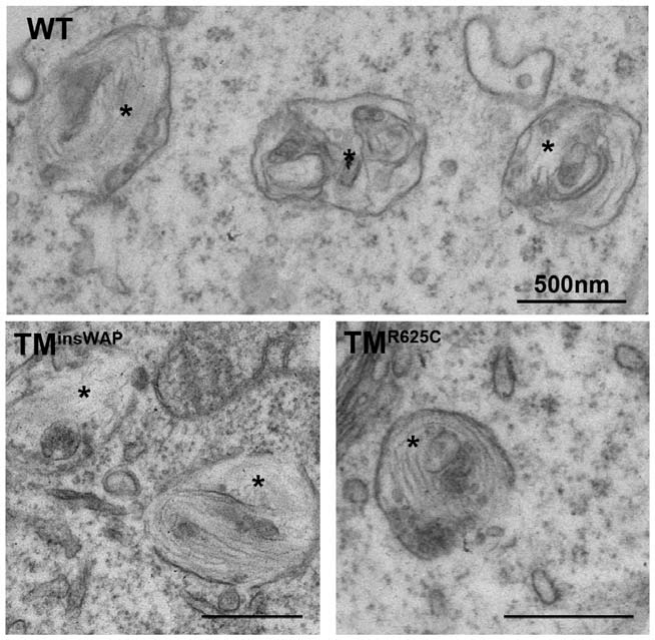
PMEL TMD mutants can form fibrils when overexpressed in non-pigmented cells. HeLa cells transiently overexpressing wild-type, TM^insWAP^ or TM^R625C^ hPMEL were fixed and embedded in epon resin for conventional electron microscopy analysis. Note that elongated fibrils in the lumen of multivesicular endocytic compartments (labeled with asterisks) are observed in cells expressing all three variants of hPMEL. Bars, 500 nm.

PMEL fibril formation is often inferred from the detection of detergent-insoluble MαC fragments by immunoblotting, but non-fibrillar mutants of PMEL that form disorganized aggregates — such as hPMEL lacking a PC cleavage site — can also generate similar fragments [12], [17]. To test whether the TM^insWAP^ and TM^R625C^ variants are capable of supporting fibril formation, HeLa cells transiently expressing these variants or wild-type hPMEL were analyzed by standard electron microscopy (EM). As shown in Figure 4, stage II-melanosome-like compartments with fibrillar arrays were detected in cells expressing any of the hPMEL variants, but not in cells expressing empty vector (Figure S2). As for cells expressing wild-type hPMEL, fibrils in cells expressing TM^insWAP^ or TM^R625C^ were detected within organelles that often contained internal membrane vesicles or sheets, but not in secretory or early endocytic compartments. Immunoelectron microscopy (IEM) analysis using immunogold labeling of ultrathin cryosections further showed that all of the hPMEL variants were incorporated into the fibrils (data not shown, but see below for incorporation of variants into melanosome fibrils in melanocytic cells). These data indicate that the TM^insWAP^ and TM^R625C^ mutations do not impair the intrinsic ability of hPMEL to form fibrils within endosomal compartments.

### TM^insWAP^ and TM^R625C^ mutations induce altered morphology and melanization of hPMEL fibrils in pigmented cells

**Figure 5:**
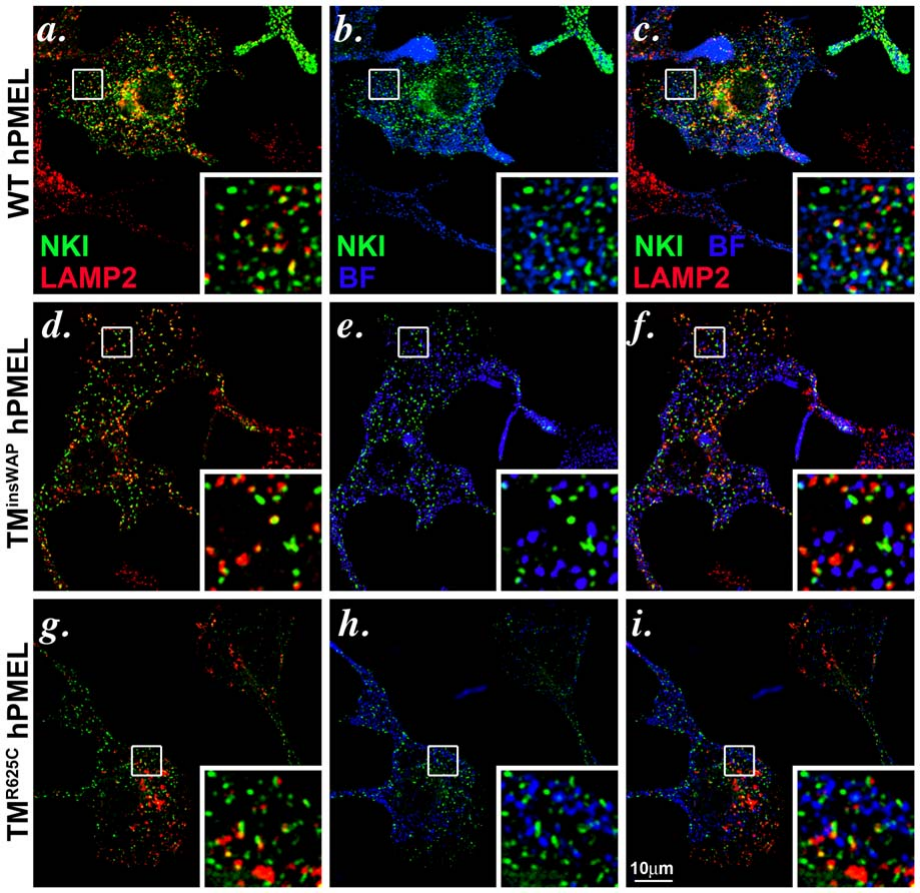
Wild-type and TMD mutant hPMEL variants localize similarly when expressed in mouse melanocytes. Immortalized melan-Ink4a mouse melanocytes that stably expressed wild-type (*a–c*), TM^insWAP^ (*d–f*) or TM^R625C^ (*g–i*) variants of hPMEL were fixed and analyzed by IFM relative to the lysosomal marker, LAMP2 (red), and to pigment granules visualized by bright field microscopy (BF; pseudocolored blue). Ectopic hPMEL was detected using NKI-Beteb (green), an antibody that recognizes human PMEL, but not murine PMEL. Shown are overlaps of PMEL relative to LAMP2 (*a*, *d*, *g*) or melanosomes (*b*, *e*, *h*) alone or together (*c*, *f*, *i*). Insets show a 4X magnification of the boxed areas. Note that both mutants show a similar pattern of staining and co-localization as wild-type hPMEL. Individual labels and the original bright field images are shown in Figure S3.

Unlike in non-pigment cells, in which fibril maturation is inefficient and occurs within late endosomes [13], PMEL protofibrils in pigment cells mature efficiently into sheets [11] that accumulate in stage II melanosomes and that then serve as sites of melanin deposition during melanosome maturation [24]. To determine whether the TM^insWAP^ and TM^R625C^ mutations influence fibril maturation or downstream pigmentation, we analyzed the behavior of hPMEL with or without these mutations expressed transiently or stably in pigment cells. In order to distinguish the transgene from the endogenous mouse PMEL, we expressed the hPMEL variants in “wild-type” mouse melanocyte cell lines, and exploited antibodies (NKI-beteb and HMB-50) that only detect hPMEL. By IFM, wild-type, TM^insWAP^ and TM^R625C^ hPMEL each localized to punctate structures that partially overlapped with late endocytic compartments marked by LAMP2 and that did not overlap with mature pigmented eumelanosomes (Figure 5 and Figure S3). These data suggest that the wild-type, TM^insWAP^ and TM^R625C^ hPMEL variants localize similarly in melanocytes. Endogenous mouse PMEL does not significantly co-localize with late endocytic markers (not shown) and thus the partial co-localization of ectopic hPMEL with LAMP2 might reflect less efficient delivery of this isoform to early stage melanosomes, likely due to the low expression levels attained by infection (see below) and the inability of hPMEL to interact with the mouse isoform as detected by coimmunoprecipitation (data not shown).

**Figure 6:**
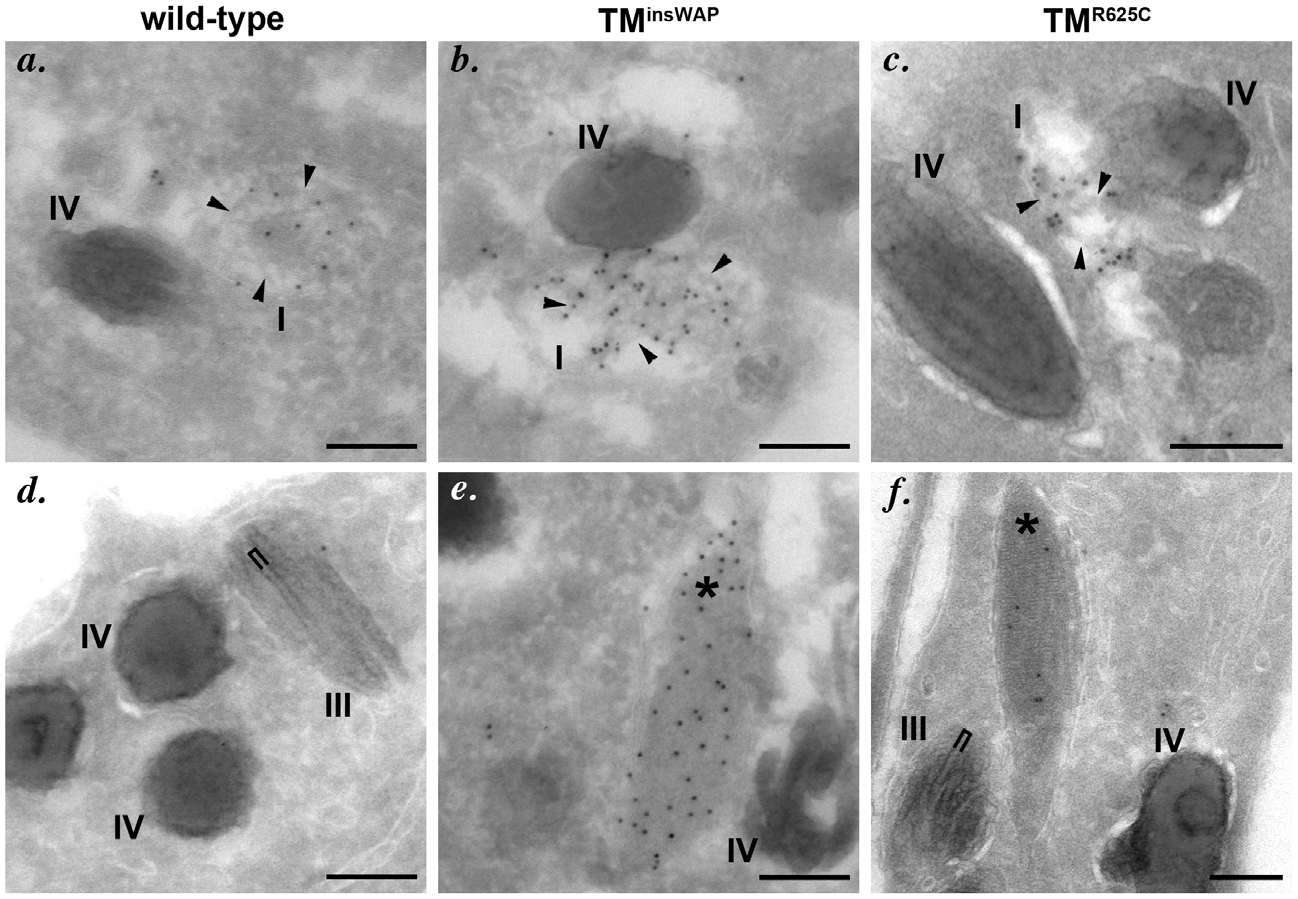
PMEL TMD mutants generate fibrils with aberrant morphology. Immortalized melan-Ink4a melanocytes that stably express wild-type (*a*, *d*), TM^insWAP^ (*b*, *e*) or TM^R625C^ (*c*, *f*) variants of hPMEL were fixed and processed for cryoimmunoelectron microscopy. Ultrathin cryosections were immunogold labeled for hPMEL using the hPMEL-specific NKI-beteb antibody and 10 nm protein A gold. Note that dense immunolabeling is observed on short immature fibrils (arrowheads) within stage I melanosomes from cells expressing all three hPMEL variants (upper panels, *a–c*), but that mature, elongated fibrils are immunogold labeled only in compartments (asterisks) within cells expressing the TMD mutants (lower panels, d–f). Also note the densely packed profile of the immunogold-labeled mature fibrils in cells expressing TMD mutant hPMEL as compared to the thick pigmented fibrils that lack labeling, which are found within stage III melanosomes in cells expressing wild-type hPMEL and which show some spacing between neighboring fibrils (bracket). Bars, 200 nm.

We next tested whether expression of the TM^insWAP^ and TM^R625C^ hPMEL variants affect the morphology or degree of pigmentation of individual melanosomes. In mouse melan-Ink4a melanocyte stable transfectants that expressed very low levels of the dominant TM^insWAP^ and TM^R625C^ hPMEL variants (∼10–20% of endogenous mouse PMEL as assessed by immunoblotting), no changes in overall pigmentation were observed relative to cells expressing wild type hPMEL (bright field images in Figure S3, with melanosomes pseudocolored blue in the insets), but this is likely due to the low expression levels (see below). Nevertheless, by IEM of ultrathin cryosections from each of the stable cell lines, thin and disorganized immature protofibrils (arrowheads) that were densely immunogold labeled with anti-hPMEL antibodies were observed within Stage I melanosomes (Figure 6a–c). However, upon maturation of the protofibrils into elongated fibrillar sheets, wild-type and mutant hPMEL showed strikingly different characteristics (compare Figure 6d to 6e–f). In cells expressing wild-type hPMEL, only very sparse immunogold labeling was observed on the parallel sheets of organized fibrils, and most of the organelles with thickened fibrillar sheets, corresponding to Stage III or IV melanosomes, were densely pigmented (Figure 6d) as has been previously reported [24]. This is consistent with the notion that although a fraction of hPMEL is delivered to the late endocytic pathway in mouse melanocytes, sufficient protein is properly trafficked to melanosomes to form fibrils. The lack of labeling on the pigmented fibrils likely reflects epitope sequestration as pigmentation proceeds [13], [14], [42]. In contrast to cells expressing wild-type hPMEL, compartments with tightly packed fibrils that were densely immunogold labeled for hPMEL were easily observed in cells expressing the TM^R625C^ and especially the TM^insWAP^ variants (asterisks, Figure 6e–f; more images can be found in Figure S4). The sheets seemed unusually tightly packed with no space between the fibrils (note the spacing between fibrils in organelles that lack labeling for the transgene, indicated by brackets, and the loss of spacing in organelles that are labeled for the TM^insWAP^ and TM^R625C^ transgenes). The organelles that were immunogold labeled largely showed no overt pigmentation, suggesting that either the fibrils were no longer capable of binding to melanins or that they inhibited melanin production. In some cases, particularly in cells expressing the TM^R625C^ variant, labeling could be observed on the non-pigmented periphery of melanized melanosomes (Figure S4h and i).

**Figure 7:**
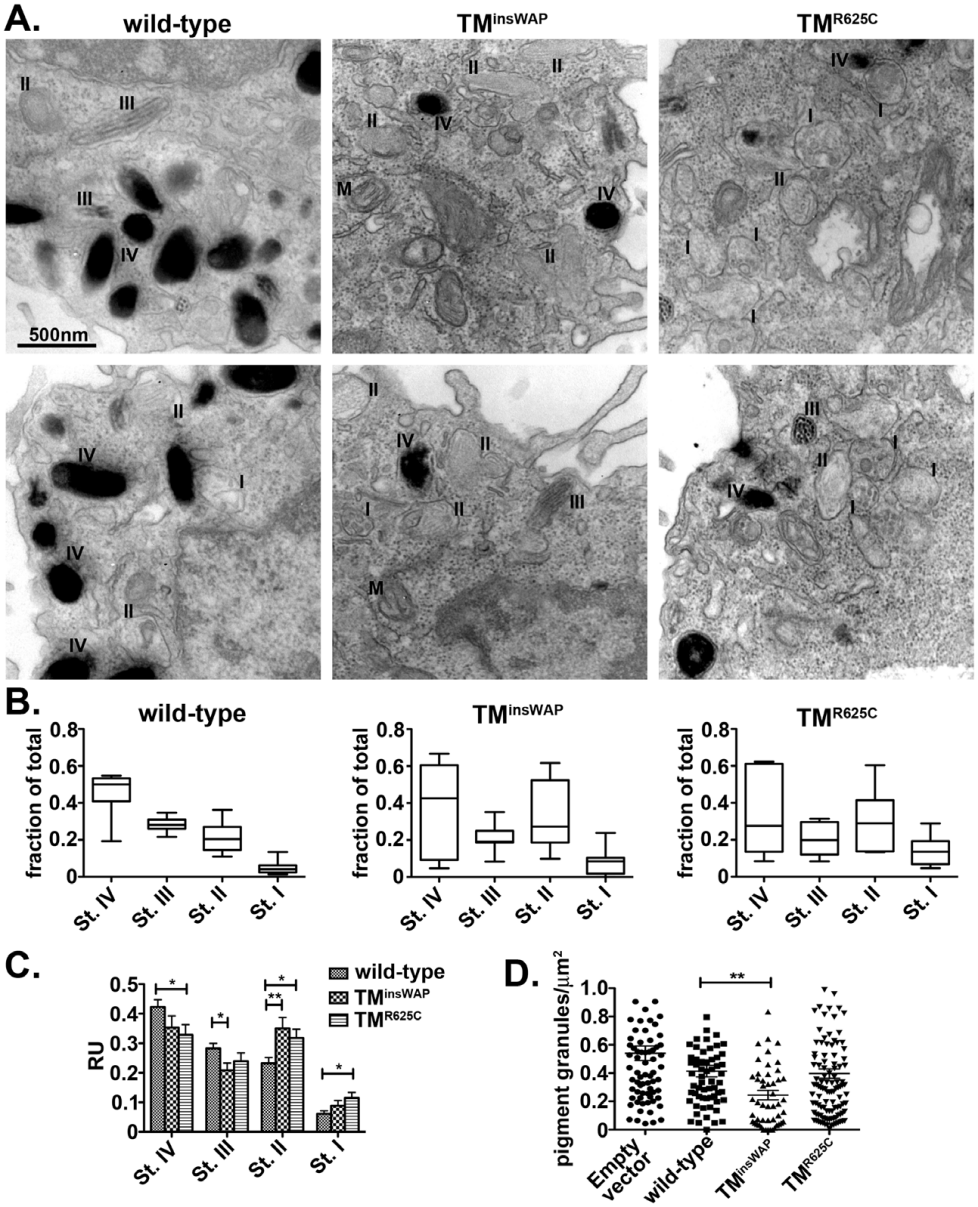
Reduced melanosome pigmentation in melanocytes expressing PMEL TMD mutants. Immortalized melanMu:MuHA pigmented melanocytes were transiently infected with retroviruses that encode wild-type, TM^insWAP^ or TM^R625C^ hPMEL-and co-expressed EGFP. Four days post-infection, cells expressing high levels of hPMEL transgene were selected by flow cytometric sorting for high EGFP expression, then fixed, embedded in epon resin, and processed for conventional thin section EM. A. Cells expressing wild-type hPMEL (left panels) show predominantly pigmented stage III and IV (III, IV) melanosomes and few unpigmented Stage I and II melanosomes (I, II). By contrast, cells expressing either TM^insWAP^ (middle panels) or TM^R625C^ (right panels) hPMEL variants harbor many fewer Stage IV melanosomes and many more non-pigmented, Stage I-II melanosomes. M, mitochondria. B. The total number of melanosomes of each stage in 6–7 whole cells from each set of samples was quantified relative to the total number of melanosomes per cell. The mean, median, maximum (max) and minimum (min) values, and 25th and 75th quartile values for distance moved in each experimental set are shown. C. Quantification of the number of each stage melanosome divided by the number of melanosomes in that field; at least 60 fields of equal size/magnification were analyzed, containing more than 400 melanosomes of different maturation stages. *, p <0.05; **, p <0.01 as determined by ANOVA and Student's t-test. D. Quantification of the number of pigmented granules (Stage III-IV) per µm^2^ of cell area in cells expressing wild-type, TM^insWAP^ or TM^R625C^ hPMEL variants or cells transduced with virus that did not co-express hPMEL. Each measurement is shown individually; median values are indicated. **, p<0.01 as determined by ANOVA and Student's t-test.

To better test whether the variant hPMEL isoforms affected overall pigmentation, we transiently expressed them in melan-mu:MuHA, a highly pigmented mouse melanocyte cell line that is less likely than melan-Ink4a to de-differentiate (our unpublished observations), using recombinant retroviruses that coexpress EGFP. The highest hPMEL expressers were enriched by cell sorting for high EGFP expression. By immunoblotting, transgene expression was substantially higher than in the stable transfectants but still less than endogenous mouse PMEL expression (data not shown). Cells were then processed for standard EM analysis. Whereas cells expressing wild-type hPMEL were normally pigmented as compared to cells expressing empty vector (not shown) and harbored few stage II (unpigmented) melanosomes, cells expressing the TM^R625C^ and especially the TM^insWAP^ variants were hypopigmented, harbored fewer pigmented melanosomes and were enriched in early stage melanosomes (Figure 7A–7B). Quantification showed that cells expressing TM^insWAP^ hPMEL showed an increase in non-pigmented Stage II (p = 0.006) with a concomitant loss of pigmented Stage III melanosomes (p = 0.014) as compared to wild-type hPMEL (Figure 7B, 7C); cells expressing TM^R625C^ hPMEL likewise showed a decrease in Stage IV melanosomes (p = 0.027) with a concomitant increase in Stage II (p = 0.018) and Stage I melanosomes (p = 0.014). Importantly, mutant PMEL-expressing cells with the highest increase in early stage melanosomes consistently showed the most marked decrease in pigmented organelles, suggesting a defect in melanosome maturation or pigment production that might be associated with higher expression levels of mutant PMEL. Moreover, quantification of the number of pigmented (Stage III and IV) organelles per unit area was decreased most strikingly in cells expressing TM^insWAP^ (p = 0.002; Figure 7D). In cells expressing TM^R625C^ there was also a tendency towards a decrease in the number of pigmented organelles (see Figure 7A–7C), but it was not significantly different from cells expressing wild-type PMEL (Figure 7D), likely due to the high variability encountered in these cells (see Figure 7B). These results suggest that even after short periods of time and with modest expression levels, the fibrils formed by the TM^insWAP^—and to a lesser degree the TM^R625C^ variant hPMEL—impair pigmentation.

Together, these results suggest that the mutations found in the dominant *DW* chicken PMEL and *Silver* horse influence the assembly of the fibrils into sheets, creating a tightly packed structure that may be inaccessible to pigment and/or that inhibits melanin biosynthesis.

### The *Smoky* chicken mutation prevents formation of aberrant PMEL fibrils

**Figure 8:**
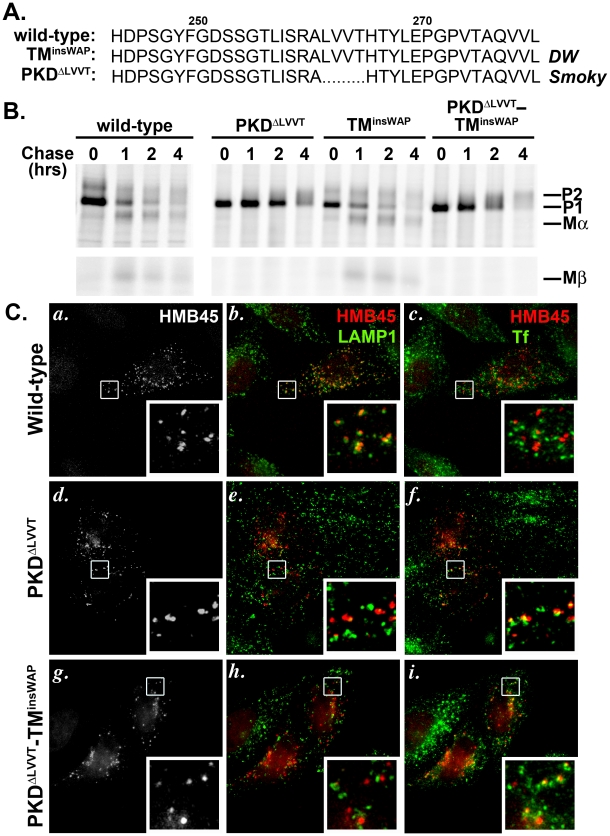
The PKD^ΔLLVT^ secondary mutation found in the *Smoky* chicken PMEL prevents delivery of TM^insWAP^ mutant hPMEL to fibrillogenic compartments. A. Sequence of PKD domain residues 245–280 in wild-type and mutant hPMEL. The PMEL gene in the *Smoky* chicken harbors an in-frame deletion that eliminates four amino acids from the PKD domain, corresponding to LVVT in hPMEL (PKD^ΔLLVT^), in addition to the TM^insWAP^ mutation. B. Metabolic labeling/pulse chase analysis of HeLa cells transiently transfected with wild-type, PKD^ΔLVVT^, TM^insWAP^ or PKD^ΔLVVT^-TM^insWAP^ variants of hPMEL. Cells were labeled, chased and Triton X-100-soluble cell lysates were immunoprecipitated with an antibody to the hPMEL C-terminus as in Figure 3C. Note that variants bearing the PKD^ΔLVVT^ deletion, regardless of the presence of the TM^insWAP^ insertion, do not mature efficiently to P2 and fail to accumulate Mα and Mβ fragments. C. IFM analysis of HeLa cells transiently expressing wild-type (*a–c*), PKD^ΔLVVT^ (*d–f*) or PKD^ΔLVVT^-TM^insWAP^ (*g–i*) variants of hPMEL. Cells were fixed after exposure for 30 min at 37°C to fluorophore-conjugated transferrin (Tf) to label early sorting and recycling endosomes, then stained with an antibody that recognizes only mature PMEL (HMB45) and an anti-LAMP1 antibody. Left panels (*a*, *d*, *g*) show PMEL labeling only; middle panels (*b*, *e*, *h*) show overlap of LAMP1 (green) and HMB45-labeled PMEL (pseudocolored red); and right panels (*c*, *f*, *i*) show overlap of internalized Tf (green) and PMEL (red). Insets show a 4X magnification of the boxed areas. Note that wild-type PMEL overlaps with LAMP1 but not internalized Tf, whereas PKD^ΔLLVT^ overlaps partially with Tf but not LAMP1.

The *Smoky* chicken is a recessive revertant of the *DW* allele, reflecting a second site mutation that results in deletion of four residues from the PKD domain (PKD^ΔLVVT^) (Figure 8A) [30]. In homozygous form, the *Smoky* allele imparts modest pigment dilution in the feathers—similar to the modest pigment dilution observed in the *silver* mouse [26], the *Pmel ^-/-^* mouse [29] and the *fading vision* zebrafish [28]—as compared to the dramatic loss of pigment imparted by the dominant *DW* allele. To investigate how the PKD^ΔLVVT^ mutation might reverse the *DW* phenotype, we created hPMEL variants with either the PKD^ΔLVVT^ deletion alone or together with the WAP insertion (PKD^ΔLVVT^-TM^insWAP^) as found in the *Smoky* chicken PMEL allele. The variants were expressed in HeLa cells, and their maturation, proteolytic processing, and trafficking were assessed by metabolic labeling/pulse chase and immunoprecipitation analysis and by IFM. As shown in Figure 8B, introduction of the PKD^ΔLVVT^ deletion, either by itself or in combination with the TM^insWAP^ insertion, impaired PMEL exit from the endoplasmic reticulum (ER) as shown by a decreased maturation into the fully glycosylated, post-Golgi P2 form. Furthermore, the PKD^ΔLVVT^ and PKD^ΔLVVT^-TM^insWAP^ hPMEL forms were not efficiently processed by proprotein convertase cleavage into Mα and Mβ fragments. These data are reminiscent of the effects of deletion of the entire PKD domain [16], [22] and suggest that the PKD^ΔLVVT^ deletion impairs PMEL maturation.

Although the pulse chase data suggested that the PKD^ΔLVVT^ mutation impairs PMEL exit from the ER, a fraction of the Golgi-modified P2 form did accumulate over time. To determine whether this fraction of “mature” PKD^ΔLVVT^ PMEL is properly trafficked to late endosomal compartments, we analyzed its localization in HeLa cells by IFM using an antibody (HMB45) that only recognizes Golgi-modified PMEL [12], [20]. Unlike wild-type hPMEL, this “mature” form of PKD^ΔLVVT^ or PKD^ΔLVVT^-TM^insWAP^ hPMEL did not localize significantly to late endosomal compartments marked by LAMP1 (Figure 8C, central panels). Rather, it partially overlapped with endocytic recycling compartments labeled by internalized transferrin (Figure 8C, right panels), much like PMEL lacking the entire PKD domain [16]. This indicates that the PKD^ΔLVVT^ deletion impairs not only PMEL maturation in the early biosynthetic pathway but also its selective incorporation into ILVs for trafficking to late endosomal compartments, thus precluding access to proprotein convertase cleavage [16]. Consistent with the requirement for sorting to ILVs and proprotein convertase cleavage for fibril formation, MαC fragments are not detected in detergent-insoluble fractions of cells expressing the PKD^ΔLVVT^ or PKD^ΔLVVT^-TM^insWAP^ mutants (Figure S5). We conclude that the PKD^ΔLVVT^ mutation reverts the dominant phenotype of the TM^insWAP^ mutation by impairing access of PMEL to fibrillogenic compartments and blocking aberrant amyloid fibril formation.

The results described above suggest that primary PMEL mutations that inhibit pigmentation in a dominant fashion can be reverted by second site mutations that prevent the aberrant PMEL variants from accumulating in fibrillogenic compartments. The dominant but milder *Dun* chicken PMEL allele contains both the orthologous TM^R625C^ mutation found in the *HoSi* allele and an additional deletion of 5 amino acids within the TMD (TM^Δ5^; Figure S6A). We therefore predicted that the TM^Δ5^ mutation, like the *Smoky*-associated PKD^ΔLVVT^ mutation, would impair PMEL accumulation within fibrillogenic endosomal compartments. Consistent with this prediction, when expressed in HeLa cells, introduction of the TM^Δ5^ mutation in the context of hPMEL decreases the expression level of all mature (post-ER) PMEL species, including the fibrillar MαC forms (Figure S6B), even when corrected for mRNA expression (data not shown). This likely reflects enhanced ER-associated degradation due to poor folding or membrane incorporation, as the material that *does* exit the ER appears to be processed with normal kinetics (Figure S6C-S6D). These data suggest that while the TM^Δ5^ mutation does not prevent delivery of PMEL to fibrillogenic compartments (Figure S6E), it can impair the efficiency with which aberrant PMEL fibrils accumulate within such compartments. Given that the primary effect of the TM^Δ5^ mutation is on ER exit, it is highly likely to have similar effects in the context of the TM^R625C^ mutation, as in *Dun* chickens, which does not affect ER exit (Figure 3).

## Discussion

Unlike most commonly known forms of amyloid, which are thought to provoke pathologic processes, PMEL is an example of a benign and functional amyloid. Here we show how mutations in the PMEL TMD are associated with an aberrant amyloid fibril biogenetic pathway, altering the normally physiological amyloid to produce a pathological form that impairs pigmentation within melanocytes. Epidermal melanocytes from animals harboring these mutations are depleted of melanosomes [34] and have decreased viability *in vitro* and perhaps *in vivo* [31], suggesting that the formation of these aberrant fibrils impairs melanosome integrity and may be toxic to the pigment cell. Although the TMD does not form part of the amyloid core, mutations in this domain influence TMD oligomeric properties that reverberate distally on the association between the amyloidogenic domains of PMEL, as evidenced by an abnormal packing of the mutant PMEL fibrils. We also show that secondary mutations found in animals in which the pigment dilution associated with the primary pathogenic TMD mutations are dampened or reverted prevent the accumulation of these PMEL isoforms in fibrillogenic compartments, thus mimicking a PMEL knockout. This finding indicates that it is less detrimental to express no fibrils at all than to express aberrant fibrils that inhibit pigmentation and might be toxic to the melanocyte.

Interactions among TMDs are known to influence multisubunit complex assembly and function in vivo [43]–[48]. Here, we show that whereas the TMD of hPMEL normally does not promote oligomerization, introduction of either the TM^insWAP^ or the TM^R625C^ mutations found in the *DW* chicken or *Silver* horse *PMEL* orthologues results in substantial oligomerization potential. Oligomerization by the PMEL TMD was similarly enhanced by insertion of three leucine residues in place of the *DW*-associated WAP insertion, suggesting that the effect reflected increased TMD length rather than specific amino acid side chain interactions. Although the observed increase in dimerization by the TM^R625C^ could not be directly compared to the wild-type TMD with the extended cytosolic domain because the latter did not insert properly into the *E. coli* plasma membrane, a similar degree of oligomerization was observed upon alteration of R^625^ to serine as with the *HoSi*-associated cysteine, but not to lysine, all of which supported proper membrane insertion. This suggests that increased oligomerization mediated by the TM^R625C^ mutation reflects removal of a positive charge from the TMD boundary, decreasing the electrostatic repulsion between neighboring PMEL molecules by the membrane proximal arginine triplet. Interestingly, the TM^R625C^ mutation was associated with greater CTF stability. In addition, whereas we often observed a CTF doublet for both wild-type and TM^insWAP^ PMEL, reflecting the two possible S2P sites [18], we always detected a single TM^R625C^ CTF species. The altered TMD mediated oligomerization of this mutant might thus result in either a greater accessibility of one site over the other or aberrant partitioning of PMEL to membrane subdomains that preferentially harbor a site-specific enzyme, akin to what has been proposed to occur between α- and β-secretases in the cleavage of APP to produce pathologic Aβ [49].

How increased TMD-mediated dimerization might influence PMEL folding, assembly, and fibril formation is not yet clear. A previous study found that the *DW* chicken PMEL associated with membrane microdomains to a similar degree as wild-type hPMEL [38], suggesting that the TM^insWAP^ mutation does not alter membrane partitioning. The same study suggested that maturation and proteolytic processing of *DW* chicken PMEL was not substantially different from that of wild-type hPMEL. Consistently, we find that neither the TM^insWAP^ nor the TM^R625C^ mutation affect hPMEL biosynthetic trafficking, proteolytic maturation, delivery to ILVs within endosomes, or the initial stages of protofibril formation. While we could not detect a previously described effect of the TM^insWAP^ mutation (or of the TM^R625C^ mutation) in reducing disulfide bond-mediated dimerization of the PMEL lumenal domain [38]—which likely reflected more a lack of heteromeric interactions between chicken and human PMEL than an effect of the TMD mutation itself—it is highly likely that the induced TMD interactions impact the orientation and proximity of PMEL dimers that form early in PMEL biosynthesis [13], [38]. Although the induced conformational changes are likely subtle and do not impact recognition by the ER quality control system, biosynthetic trafficking, or the ability to form fibrils, they do appear to have downstream effects on the assembly of fibrils into sheets and/or in the packing of the sheets. One potential explanation for these effects is that non-amyloidogenic domains of PMEL dimers that protrude from the fibrils and regulate the packing of fibrils into higher order assemblies might be positioned differently. An alternative explanation is that oligomerization via the PMEL TMD might increase the kinetics of higher order fibril assembly. Either effect might result in more tightly packed fibrils within early stage melanosomes.

How might the TM^insWAP^ or TM^R625C^ PMEL variants impair melanogenesis and melanosome integrity? If indeed increased TMD oligomerization translates a conformational change to the lumenal domain to alter either the mode or kinetics of fibril polymerization into sheets, several mechanisms could be envisioned. Both altered conformation or kinetics—either by physical blockade through tighter packing or by overly rapid kinetics of sheet assembly—would potentially preclude the delivery of melanogenic enzymes, such as tyrosinase, to the lumen of the maturing melanosome [50]. This would in turn have the effect of concentrating the formation of oxidative melanin intermediates at the limiting membrane of the maturing melanosome and subjecting the limiting membrane to oxidative attack, potentially damaging the integrity of the organelle. This would explain both the loss of melanization (this study and refs. [31], [34]), despite the presence of a potentially active tyrosinase [34], [35], and the decrease in melanosome numbers [34], [35]. Release of melanosomal contents might then impact cell viability [31]. Alternatively, it is known that melanosomes are highly enriched in divalent cations [51]–[53], and PMEL has been suggested to sequester calcium [54]; alterations to PMEL fibril packing might reduce its ability to sequester divalent cations, with potential harmful effects on melanosomes by further oxidative damage. Potential negative effects on copper-dependent tyrosinase activity within melanosomes might result from a similar loss of copper sequestration [55]. A third possibility is that the altered conformation of the fibrils—which very likely are a variant form of amyloid—makes them inherently toxic. For example, Aβ amyloid has been shown to insert into and disrupt lipid bilayers [56]; a similar property of the TM^insWAP^ and TM^R625C^ PMEL amyloid fibrils could potentially disrupt the melanosome membrane directly, leading to a loss of melanosome integrity and consequent loss of pigmentation. Finally, it is possible that pigmentation and melanosome viability are disrupted by an intermediate in fibril or sheet assembly that might persist due to a decrease in kinetics or that might be produced only by the variants. None of these possibilities are mutually exclusive, and it is possible that a combination of effects inhibits pigmentation.

It has not been understood why the phenotype of the *Smoky* chicken, with a PKD^ΔLVVT^ mutation in addition to the *DW*-associated TM^insWAP^ mutation, restores substantial pigmentation relative to the parental *DW* chicken [30]. We show here that the secondary PKD^ΔLVVT^ mutation prevents the accumulation of PMEL in fibrillogenic compartments, likely explaining the decreased pigment dilution observed in *Smoky* vs. *DW* chickens. The PKD^ΔLVVT^ mutation largely impairs PMEL maturation through the early biosynthetic pathway, causing retention in the ER. Moreover, the small fraction of PMEL that exits the ER is not selectively targeted to multivesicular endosomes and rather accumulates in early endosomal recycling compartments. This correlates with a lack of proprotein convertase cleavage into Mα and Mβ fragments and decreased accumulation of PMEL fragments in detergent insoluble, fibril-enriched fractions. The behavior of this mutant form of hPMEL is similar to that of hPMEL in which the entire PKD domain is deleted [16], supporting a critical role for the PKD domain in targeting PMEL to fibrillogenic compartments and perhaps directly in fibrillogenesis [23]. The results indicate that the *Smoky* allele is functionally a PMEL null allele that counteracts the *DW* mutant's pathogenic effects on pigmentation in a recessive manner by preventing the formation of aberrant fibrils. Thus, *Smoky* chickens show a slight pigment dilution similar to that observed in PMEL knockout mice [29] or in the hypomorphic *silver* mouse [26], [33] rather than a dramatic loss of eumelanin pigment as observed in the *DW* chicken and *Silver* horse. Since melanocytes in the *DW* chicken show decreased viability [31] and melanosome integrity [34], our studies further suggest that the formation of tightly packed TM^insWAP^ fibrils may be toxic to pigment cells and that it is therefore less detrimental to the cell to have no PMEL fibrils at all.

The reversion of the dominant *DW* phenotype by the PKD^ΔLVVT^ mutation in *Smoky* chickens suggests that a general mechanism for averting PMEL amyloid pathology is to prevent access of the aberrant amyloidogenic protein to compartments within which amyloid formation occurs. The *Dun* chicken appears to be another example of such a mechanism. Whereas the *Dun* chicken PMEL allele contains a mutation orthologous to that of the dominant TM^R625C^ mutation in the toxic *Silver* horse PMEL, it also has a secondary deletion of 5 amino acids in the TMD (TM^Δ5^) [30]. Introduction of this secondary mutation into hPMEL impairs ER exit, trafficking through the plasma membrane (data not shown), and accumulation of all mature PMEL species at steady state, suggesting inefficient PMEL folding and a greater propensity for degradation. Thus, while the mechanism is different from that of the secondary PKD^ΔLVVT^ mutation in *Smoky* chickens, the overall effect of the TM^Δ5^ mutation in *Dun* chickens might be similar — a reduction of aberrant PMEL accumulation in fibrillogenic compartments. We therefore liken this secondary mutation to a revertant of the *Silver* horse phenotype. We speculate that mutations in other toxic/ pathological forms of amyloidogenic proteins that prevent appropriate accumulation of the amyloidogenic species within amyloidogenic compartments will be associated with protection from disease.

## Materials and Methods

### Antibodies and reagents

The mouse monoclonal antibodies used, their targets and sources were as follows: HMB45 and NKI-Beteb to PMEL were from Lab Vision (Freemont, CA); TA99 to TYRP1 was from American Type Culture Collection (Manassas, VA); H4A3 to LAMP1 was from Developmental Studies Hybridoma Bank (University of Iowa, Iowa City, IA). The rabbit polyclonal antibodies used, their targets and sources were αPep13h to the C-terminal peptide of hPMEL [13], αPmel-N to the N-terminal peptide of hPMEL [17], α-LAMP1 from Affinity BioReagents (Golden, CO), and α-MBP from New England Biolabs (Beverly, MA). Rat α-LAMP2 was from Developmental Studies Hybridoma Bank. Unless otherwise specified, chemicals were obtained from Sigma-Aldrich (St. Lois, MO). Tissue culture reagents were from Invitrogen (Carlsbad, CA). FuGENE-6 and hygromycin were from Roche Diagnostics (Indianapolis, IN).

### DNA constructs and cloning

Wild-type hPMEL (long form) in pCI has been described [13] and was used as a template for site directed mutagenesis via PCR to generate the TM^insWAP^, TM^R625C^, PKD^ΔLVVT^, PKD^ΔLVVT^-TM^insWAP^, TM^Δ5^ mutations; the primers used are indicated in Table S1. Wild-type and mutant hPMEL XhoI-NotI inserts from pCI were subcloned into pBMN-IRES-Hygro (a gift from R. Scheller, Genentech, San Francisco, CA) or pBMN-IRES-EGFP retroviral vectors for stable or transient infection, respectively. For the TOXCAT assays, the pccKan expression vector was used. The pccGpA WT and pccGpA G83I mutant derivatives of pccKan, encoding wild-type or mutant glycophorin A, as well as the MBP deficient *Escherichia coli* (*malE^-^*) have been described [39]. PMEL wild-type or mutant TMDs were PCR-amplified from their pCI templates and cloned into the NheI-BamHI sites of pccKan. All plasmid inserts were verified by DNA sequencing.

### Maltose complementation and TOXCAT

*malE^-^* bacteria were transformed with pccKan or its derivatives encoding the ToxR-TMD-MBP chimeras. Ampicillin resistant colonies were selected, grown to mid-log phase in Luria Broth, streaked over amp-M9 agar plates with either glucose (positive control) or maltose as the only source of carbon [39], and incubated for 2–4 days at 37°C. CAT activity of clones that grew in maltose was measured using a spectrophotometric assay [40]. Briefly, the pellet from 1 ml of OD600 = 0.75 culture was resuspended in 300 µl of sonication buffer (25 mM Tris pH 7.8 2 mM EDTA), sonicated, and centrifuged 30 min at 4°C, 13000 x g to obtain cell free extracts. 10 µl of lysate was combined with 230 µl of CAT reaction buffer (100 mM Tris pH 7.8, 0.4 mg/ml DTNB, 0.1 M AcetylCoA) and the A^412 nm^ was recorded every minute for 5 min to obtain the background. Then, 10 µl of 2.5 mM chloramphenicol was added to initiate the CAT reaction and the A^412 nm^ was recorded every minute for 30 min to determine the CAT activity according to the method by Shaw [40]. The change in A^412 nm^ was linear throughout the experiment.

### Cell culture and transgene expression

HeLa cells were grown as described previously [13] and transiently transfected with 0.1 µg DNA/ 3-cm dish for low or 7.5 µg DNA/ 10-cm dish for high transgene expression using FuGENE-6 according to the manufacturer's instructions. HeLa cells were analyzed either 48 hrs (immunofluorescence microscopy, metabolic labeling/pulse chase and immunoblotting) or 72–96 hrs (electron microscopy) post-transfection. The immortalized mouse melanocyte cell lines melan-Ink4a [57] and melan-mu:MuHA rescued cells (MuHA; [58]) were grown as described previously [57] and stably or transiently infected with the retroviral vectors described above. Stable melan-Ink4a transductants were selected with 200–400 µg hygromycin B and processed for immunofluorescence (IFM) or immunoelectron microscopy (IEM). Transiently infected MuHA cells were sorted (University of Pennsylvania Cell Sorting Core Facility, Philadelphia, PA) for high EGFP expression 96 hrs post-infection and processed for conventional electron microscopy (EM).

### Immunofluorescence microscopy

Cells were fixed with 2% formaldehyde, incubated with primary and fluorochrome-conjugated secondary antibodies as described previously [13] and analyzed on a DM IRBE microscope (Leica Microsystems, Wetzlar, Germany). Digital images were captured with an Orca camera (Hamamatsu, Bridgewater, NJ) and deconvolved and manipulated with OpenLab software (Improvision, Lexington, MA). Insets were magnified using Adobe Photoshop (Adobe Systems, Mountain View, CA). For recycling endosome labeling by continuous transferrin (Tf) uptake, HeLa cells were starved in serum-free media containing 0.5% BSA 15 mM HEPES for 30 min at 37°C, incubated with 7.5 µg/ml Alexafluor488-conjugated human Tf (Molecular Probes, Eugene, OR) diluted in starvation media for an additional 30 min at 37°C, fixed, and processed for IFM as indicated above.

### Electron microscopy

For conventional electron microscopy, cells were fixed in 2% glutaraldehyde 4% paraformaldehyde, dehydrated and embedded in epon resin. Ultrathin sections were contrasted with 2% uranyl acetate and analyzed by transmission electron microscopy. For immunoelectron microscopy (IEM), cells were fixed with 2% paraformaldehyde 0.5% glutaraldehyde and ultrathin frozen sections were single- or double-immunogold labeled as described previously [14], [59] using Protein A gold conjugates. For quantification of numbers of Stage I-IV melanosomes, at least 50 random fields of each cell type (>400 compartments) were analyzed.

### Immunoblotting, metabolic labeling/pulse chase, and immunoprecipitation analyses

For immunoblotting, cells were harvested with 5 mM EDTA in PBS, washed with 30 mM NEM in PBS, and frozen. Thawed cells were lysed with TX-100 lysis buffer as described previously [60] in the presence of protease inhibitors and NEM and fractionated into detergent soluble and insoluble fractions by centrifugation. For metabolic labeling, cells were harvested with trypsin-EDTA, starved for 30 min in methionine/cysteine free media, labeled with ^35^S methionine-cysteine for 30 min and chased for the indicated periods of time. Cells were then lysed as indicated above and detergent soluble fractions were immunoprecipitated with antibodies directed to the C-terminus of hPMEL, followed by SDS-PAGE fractionation and analysis with a STORM PhosphorImager and ImageQuant software (GE Healthcare, Buckinghamshire, United Kingdom).

## Supporting Information

Figure S1 TMD mutations do not influence hPMEL covalent oligomerization and expression of TMD mutant chimeras in *E. coli*. A. HeLa cells transiently transfected with wild-type (wt), TM^insWAP^ or TM^R625C^ variants of hPMEL were lysed, and Triton X-100 soluble fractions were fractionated by SDS-PAGE in the absence (-2ME) or presence (+2ME) of 2-mercaptoethanol. Immunoblots were probed with an antibody to the N-terminus of hPMEL. Note that the three variants migrate identically under both non-reducing and reducing conditions, indicating that each variant is capable of generating appropriate interchain disulfide bonds. B. Whole cell lysates from cultures of bacteria that were either untransformed (untrfm.) or transformed to express the indicated ToxR- PMEL-TM-MBP chimeric proteins were fractionated by SDS-PAGE and probed with anti-MBP antibody (IB: α-MBP). Note that all fusion proteins were expressed at similar levels.
(TIF)

Figure S2 Absence of fibrillar compartments in HeLa cells that do not express PMEL variants. HeLa cells transiently transfected with empty pCI vector were fixed and embedded in epon resin for conventional electron microscopy analysis, as in Figure 4. Note the absence of fibrillar structures that are observed in cells expressing either wild-type or TMD variant forms of hPMEL (Figure 4) and the presence of numerous multivesicular/ multilaminar, electron dense lysosomes (L). M, mitochondria. Bar, 500 nm.
(TIF)

Figure S3 Localization of wild-type, TM^insWAP^ and TM^R625C^ hPMEL variants relative to lysosomes and pigment granules in melanocytes. Shown are individual panels of the overlays shown in Figure 5 for immortalized melan-Ink4a cells stably expressing wild-type (WT; top panels), TM^insWAP^ (middle panels) or TM^R625C^ (bottom panels) variants of hPMEL. See legend to Figure 5 for details. Left, labeling for hPMEL variants only (green). Middle, labeling for LAMP2 only (red). Right, bright field images of pigment granules only. Insets, 4X magnifications of boxed regions, showing overlap of hPMEL variant (green) with LAMP1 (red; left panels), pigment granules (pseudocolored blue; middle panels), or with both (right panels). Bar, 10 µm.
(TIF)

Figure S4 PMEL TMD mutants form aberrantly packed fibrils within pigmented cells. Immortalized melan-Ink4a melanocytes that stably express wild-type (*a*–c), TM^insWAP^ (*d–f*) or TM^R625C^ (*g–i*) variants of hPMEL were fixed and processed for cryoimmunoelectron microscopy. Ultrathin cryosections were immunogold labeled for hPMEL using the hPMEL-specific NKI-beteb antibody and 10 nm protein A gold; shown are additional images to complement those in Figure 6. Note the presence of aberrantly packed, unpigmented compartments (asterisks) that are densely labeled with antibodies against hPMEL only in cells expressing the TMD mutant isoforms. Melanosomes of stage I, II, III and IV are indicated. Scale bar, 0.2 µm except in panel *a*, in which it represents 0.5 µm.
(TIF)

Figure S5 PKD^ΔLVVT^ deletion found in *Smoky* chickens eliminates the formation of detergent-insoluble MαC fragments by hPMEL. HeLa cells transiently transfected with wild-type (wt), TM^insWAP^, PKD^ΔLVVT^, PKD^ΔLVVT^ –TM^insWAP^, or TM^R625C^ variants of hPMEL were lysed and fractionated into detergent soluble (S) and insoluble (I) fractions. Identical cell equivalents of each fraction were separated by SDS-PAGE and analyzed by immunoblotting with HMB45 anti-PMEL antibody. Left, molecular weight markers; right, full-length Mα and fibril-associated MαC fragments are indicated. Note the absence of HMB45-reactive bands in cells expressing the PKD^ΔLVVT^ and PKD^ΔLVVT^ –TM^insWAP^ variants.
(TIF)

Figure S6 The TM^Δ5^ mutation found in *Dun* chickens impairs hPMEL maturation. A. Sequence of TMD residues 596-627 in wild-type and TM^Δ5^ variant hPMEL. The PMEL gene in the *Dun* chicken harbors an in-frame deletion that eliminates five amino acids from the middle of the TMD, in addition to the TM^R625C^ mutation that is also found in the *Silver* horse PMEL. B. Immunoblot analysis. Transfected HeLa cells expressing wild-type (wt) or TM^Δ5^ hPMEL variants were lysed and fractionated into detergent soluble and insoluble fractions. Detergent soluble fractions (top three panels) were probed with antibodies to either the hPMEL N-terminus to detect P1 and Mα (top), or the C-terminus to detect Mβ and CTF (middle panels). Detergent insoluble, fibril-enriched fractions were probed with HMB45 to detect the PMEL-derived MαC fragments (lower panel). Left, molecular weight markers; right, relevant bands are indicated. C. Metabolic labeling/pulse chase analysis of HeLa cells transiently transfected with wild-type or TM^Δ5^ variant hPMEL. Cells were labeled, chased and Triton X-100-soluble cell lysates were immunoprecipitated with antibody to the hPMEL C-terminus as in Figure 3C. D. Quantification of abundance of relevant PMEL fragments from the pulse/ chase experiment shown in C.; the band intensity of each fragment was normalized to that of P1 at time zero [P1(t_0_)]. Note the reduced fraction of all TM^Δ5^ post-ER bands. E. IFM analysis of HeLa cells transiently expressing TM^Δ5^ variant hPMEL. Cells were labeled with NKI-beteb monoclonal antibody to PMEL (left, red) and with anti-LAMP1 antibody (overlay shown on the right). Insets show a 4X magnification of the boxed region. Note the presence of TM^Δ5^ variant hPMEL within structures circled by LAMP1, as observed for wild-type hPMEL (see Figure 3A).
(TIF)

Table S1 Primers used to make full-length hPMEL mutants.
(DOC)
