# Supplementary figures and images for: Mutations in or near the Transmembrane Domain Alter PMEL Amyloid Formation from Functional to Pathogenic
Source: PLoS Genet. 2011 Sep 15;7(9):e1002286. doi: 10.1371/journal.pgen.1002286 (PMC3174235; doi:10.1371/journal.pgen.1002286)

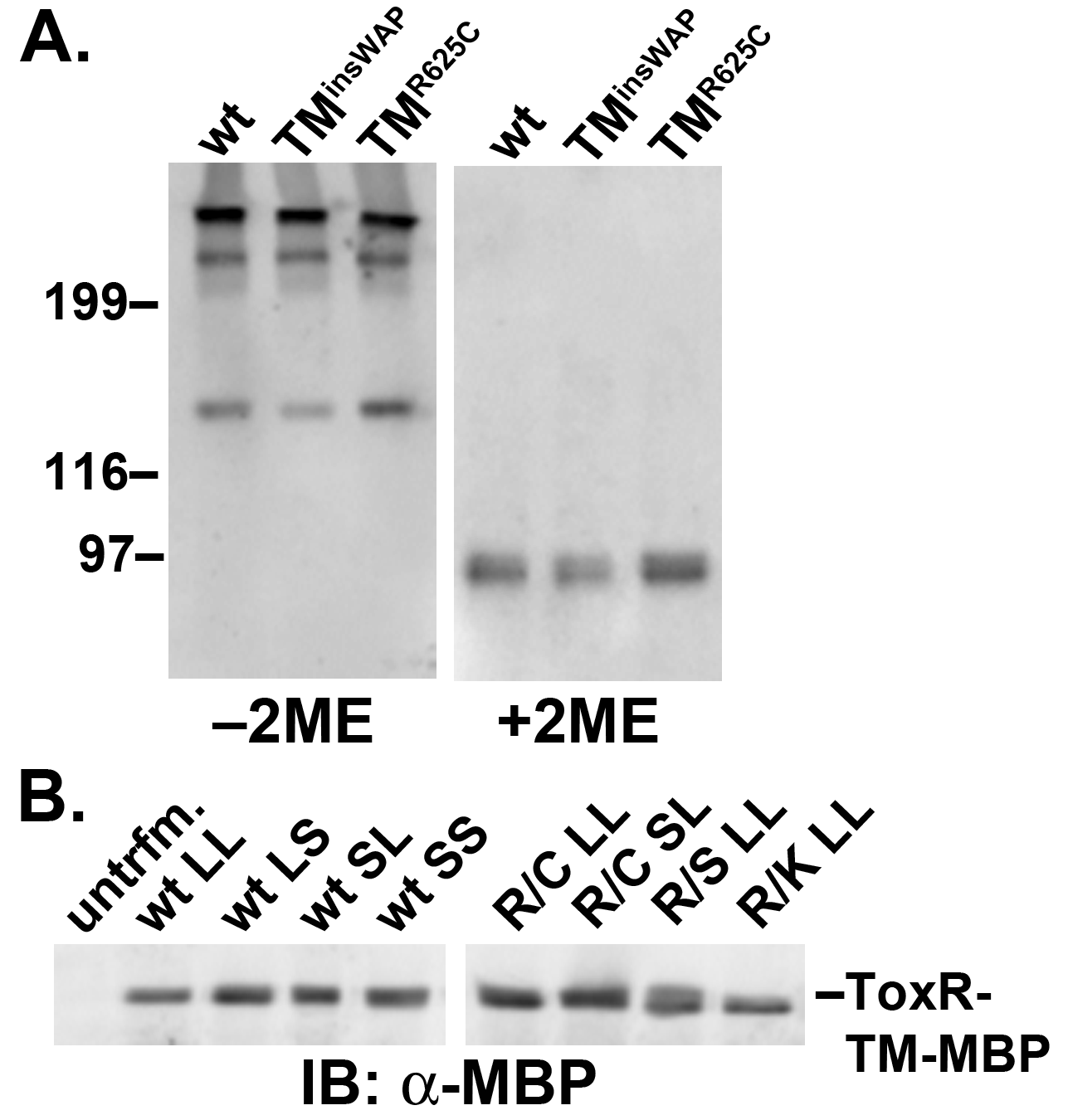

Supplement: Figure S1 — TMD mutations do not influence hPMEL covalent oligomerization and expression of TMD mutant chimeras in E. coli. A. HeLa cells transiently transfected with wild-type (wt), TMinsWAP or TMR625C variants of hPMEL were lysed, and Triton X-100 soluble fractions were fractionated by SDS-PAGE in the absence (-2ME) or presence (+2ME) of 2-mercaptoethanol. Immunoblots were probed with an antibody to the N-terminus of hPMEL. Note that the three variants migrate identically under both non-reducing and reducing conditions, indicating that each variant is capable of generating appropriate interchain disulfide bonds. B. Whole cell lysates from cultures of bacteria that were either untransformed (untrfm.) or transformed to express the indicated ToxR- PMEL-TM-MBP chimeric proteins were fractionated by SDS-PAGE and probed with anti-MBP antibody (IB: α-MBP). Note that all fusion proteins were expressed at similar levels. (TIF) [file pgen.1002286.s001.tif]

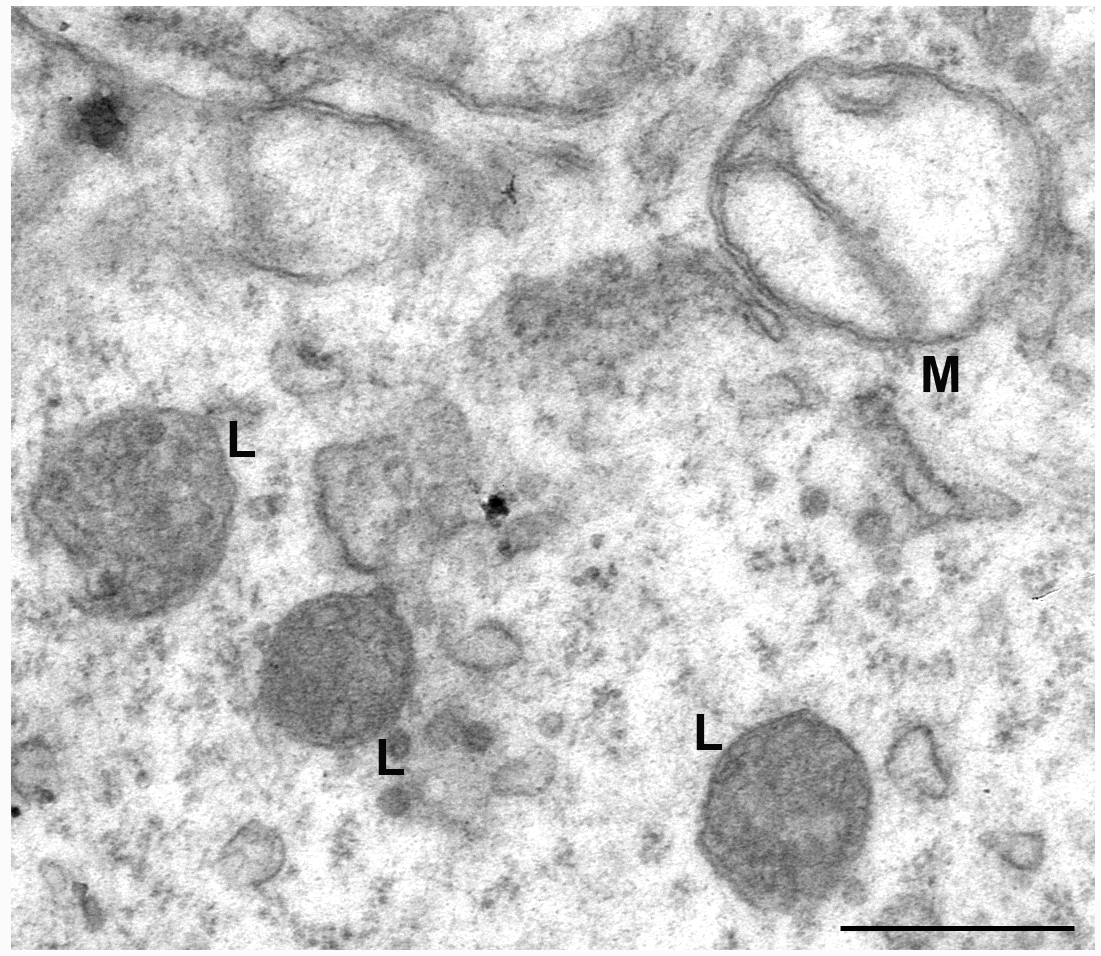

Supplement: Figure S2 — Absence of fibrillar compartments in HeLa cells that do not express PMEL variants. HeLa cells transiently transfected with empty pCI vector were fixed and embedded in epon resin for conventional electron microscopy analysis, as in Figure 4. Note the absence of fibrillar structures that are observed in cells expressing either wild-type or TMD variant forms of hPMEL (Figure 4) and the presence of numerous multivesicular/ multilaminar, electron dense lysosomes (L). M, mitochondria. Bar, 500 nm. (TIF) [file pgen.1002286.s002.tif]

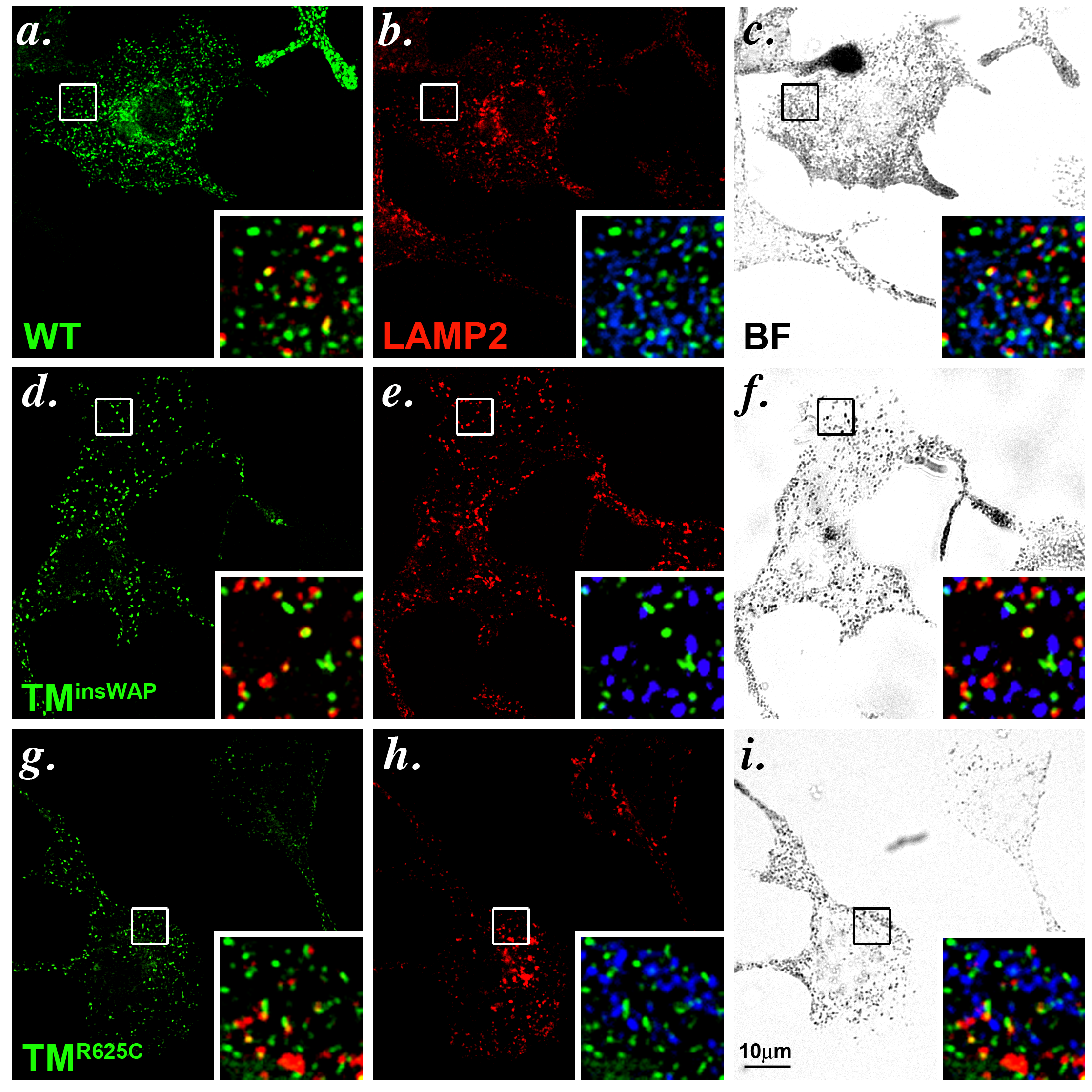

Supplement: Figure S3 — Localization of wild-type, TMinsWAP and TMR625C hPMEL variants relative to lysosomes and pigment granules in melanocytes. Shown are individual panels of the overlays shown in Figure 5 for immortalized melan-Ink4a cells stably expressing wild-type (WT; top panels), TMinsWAP (middle panels) or TMR625C (bottom panels) variants of hPMEL. See legend to Figure 5 for details. Left, labeling for hPMEL variants only (green). Middle, labeling for LAMP2 only (red). Right, bright field images of pigment granules only. Insets, 4X magnifications of boxed regions, showing overlap of hPMEL variant (green) with LAMP1 (red; left panels), pigment granules (pseudocolored blue; middle panels), or with both (right panels). Bar, 10 µm. (TIF) [file pgen.1002286.s003.tif]

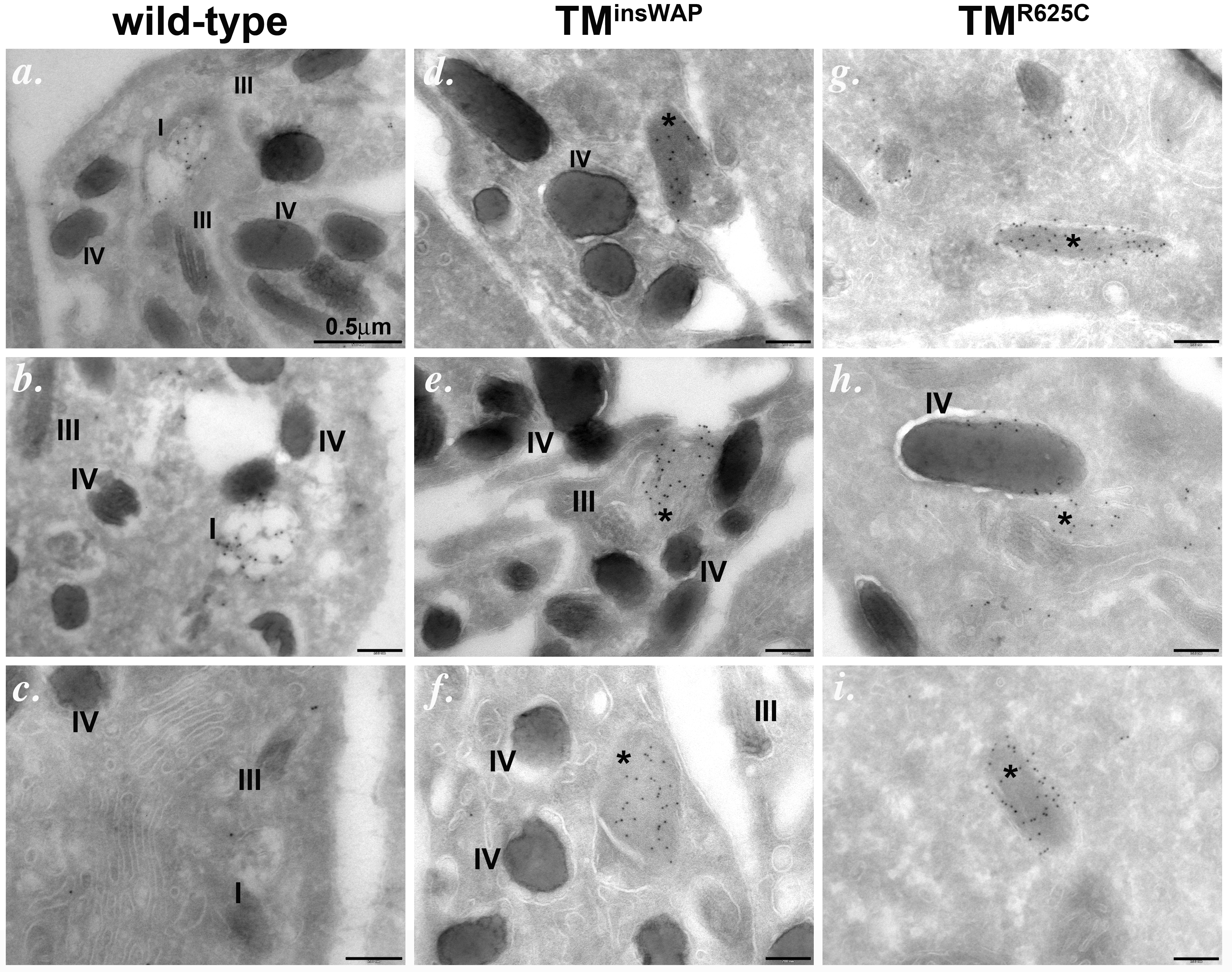

Supplement: Figure S4 — PMEL TMD mutants form aberrantly packed fibrils within pigmented cells. Immortalized melan-Ink4a melanocytes that stably express wild-type (a–c), TMinsWAP (d–f) or TMR625C (g–i) variants of hPMEL were fixed and processed for cryoimmunoelectron microscopy. Ultrathin cryosections were immunogold labeled for hPMEL using the hPMEL-specific NKI-beteb antibody and 10 nm protein A gold; shown are additional images to complement those in Figure 6. Note the presence of aberrantly packed, unpigmented compartments (asterisks) that are densely labeled with antibodies against hPMEL only in cells expressing the TMD mutant isoforms. Melanosomes of stage I, II, III and IV are indicated. Scale bar, 0.2 µm except in panel a, in which it represents 0.5 µm. (TIF) [file pgen.1002286.s004.tif]

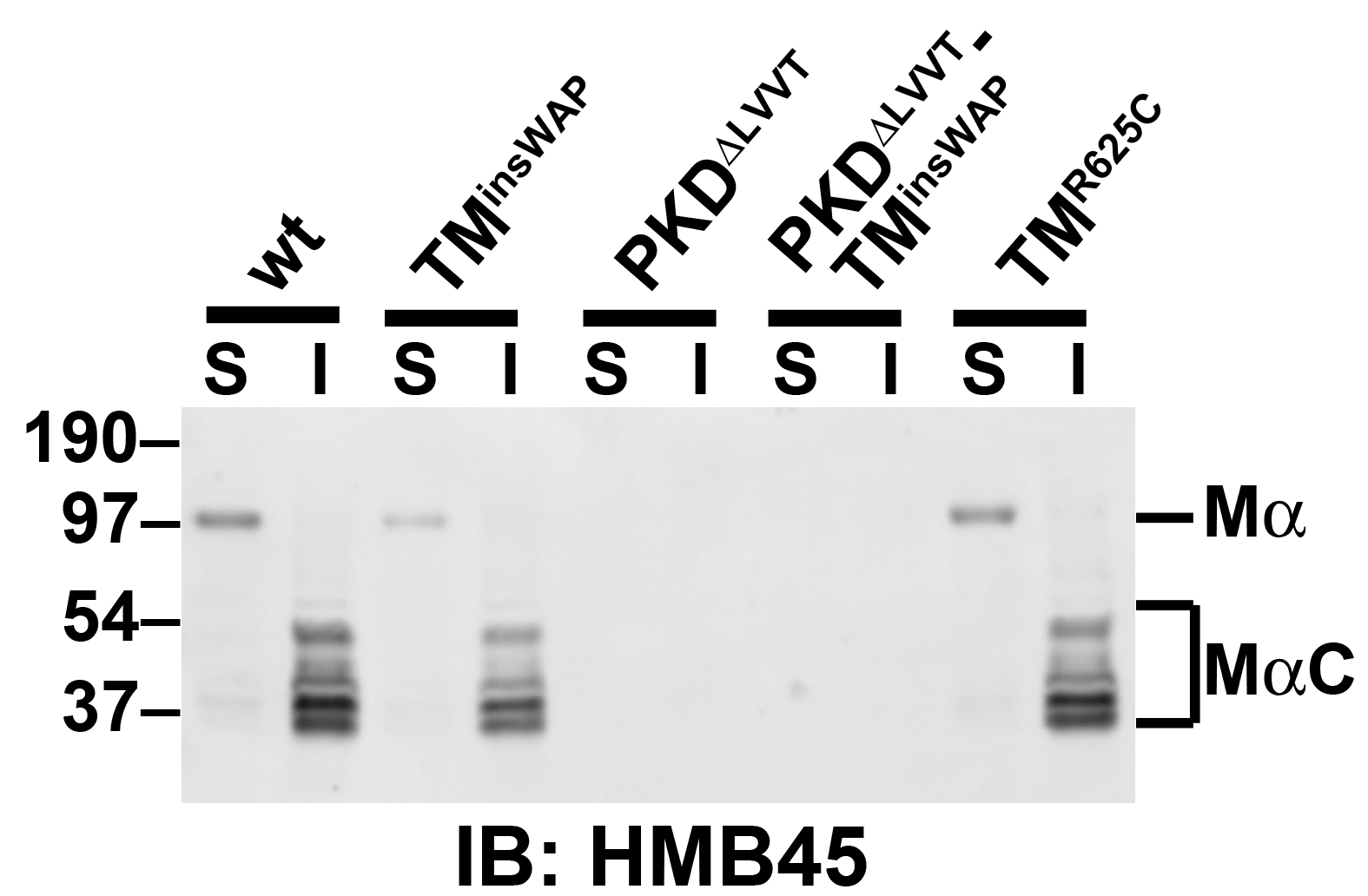

Supplement: Figure S5 — PKDΔLVVT deletion found in Smoky chickens eliminates the formation of detergent-insoluble MαC fragments by hPMEL. HeLa cells transiently transfected with wild-type (wt), TMinsWAP, PKDΔLVVT, PKDΔLVVT –TMinsWAP, or TMR625C variants of hPMEL were lysed and fractionated into detergent soluble (S) and insoluble (I) fractions. Identical cell equivalents of each fraction were separated by SDS-PAGE and analyzed by immunoblotting with HMB45 anti-PMEL antibody. Left, molecular weight markers; right, full-length Mα and fibril-associated MαC fragments are indicated. Note the absence of HMB45-reactive bands in cells expressing the PKDΔLVVT and PKDΔLVVT –TMinsWAP variants. (TIF) [file pgen.1002286.s005.tif]

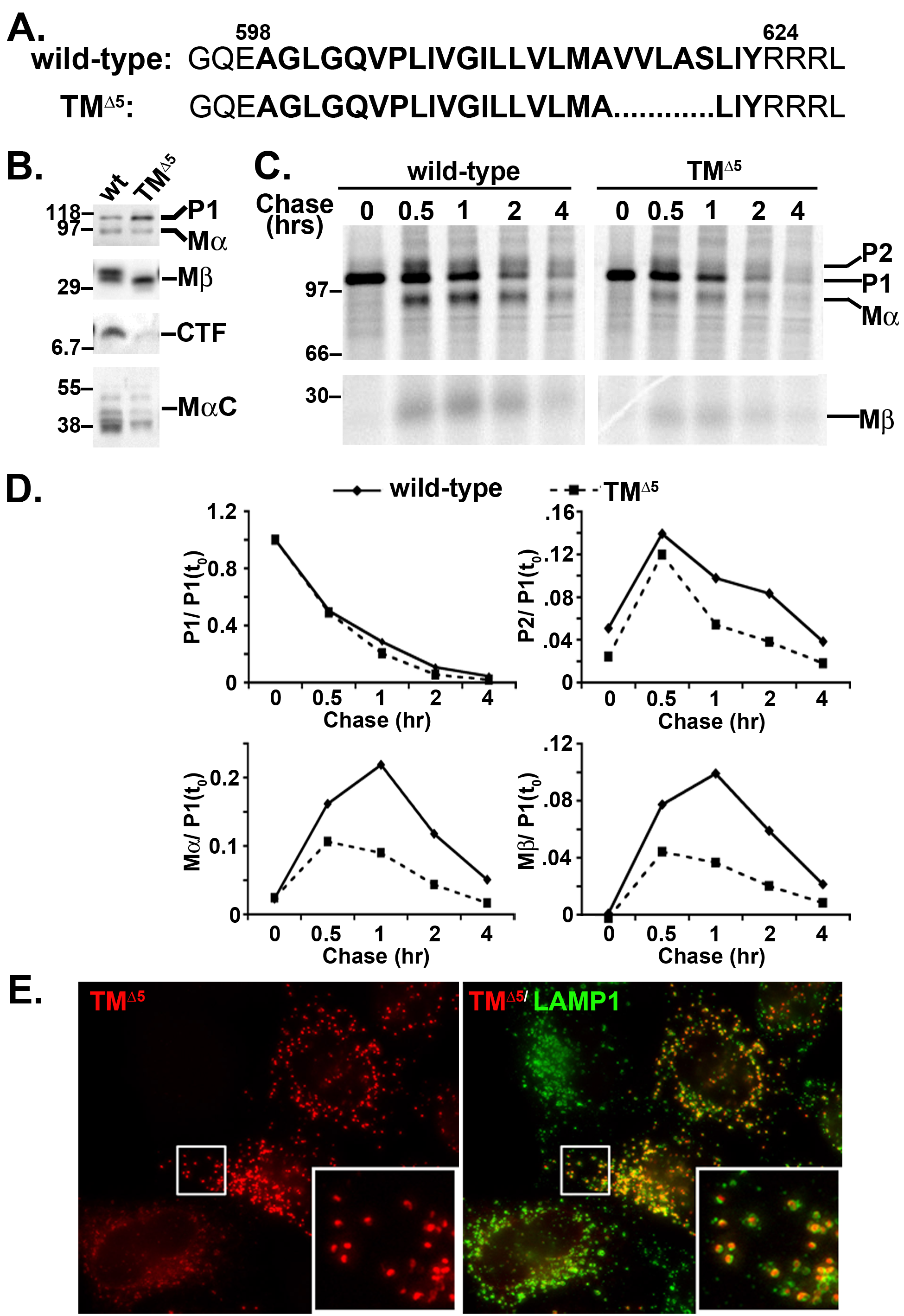

Supplement: Figure S6 — The TMΔ5 mutation found in Dun chickens impairs hPMEL maturation. A. Sequence of TMD residues 596-627 in wild-type and TMΔ5 variant hPMEL. The PMEL gene in the Dun chicken harbors an in-frame deletion that eliminates five amino acids from the middle of the TMD, in addition to the TMR625C mutation that is also found in the Silver horse PMEL. B. Immunoblot analysis. Transfected HeLa cells expressing wild-type (wt) or TMΔ5 hPMEL variants were lysed and fractionated into detergent soluble and insoluble fractions. Detergent soluble fractions (top three panels) were probed with antibodies to either the hPMEL N-terminus to detect P1 and Mα (top), or the C-terminus to detect Mβ and CTF (middle panels). Detergent insoluble, fibril-enriched fractions were probed with HMB45 to detect the PMEL-derived MαC fragments (lower panel). Left, molecular weight markers; right, relevant bands are indicated. C. Metabolic labeling/pulse chase analysis of HeLa cells transiently transfected with wild-type or TMΔ5 variant hPMEL. Cells were labeled, chased and Triton X-100-soluble cell lysates were immunoprecipitated with antibody to the hPMEL C-terminus as in Figure 3C. D. Quantification of abundance of relevant PMEL fragments from the pulse/ chase experiment shown in C.; the band intensity of each fragment was normalized to that of P1 at time zero [P1(t0)]. Note the reduced fraction of all TMΔ5 post-ER bands. E. IFM analysis of HeLa cells transiently expressing TMΔ5 variant hPMEL. Cells were labeled with NKI-beteb monoclonal antibody to PMEL (left, red) and with anti-LAMP1 antibody (overlay shown on the right). Insets show a 4X magnification of the boxed region. Note the presence of TMΔ5 variant hPMEL within structures circled by LAMP1, as observed for wild-type hPMEL (see Figure 3A). (TIF) [file pgen.1002286.s006.tif]
