# Supplementary material for: Mutations in or near the Transmembrane Domain Alter PMEL Amyloid Formation from Functional to Pathogenic
Source: PLoS Genet. 2011 Sep 15;7(9):e1002286. doi: 10.1371/journal.pgen.1002286 (PMC3174235; doi:10.1371/journal.pgen.1002286)
Supplement: Table S1 — Primers used to make full-length hPMEL mutants. (DOC) [file pgen.1002286.s007.doc]

**Table S1.** Primers used to make full-length hPMEL mutants.

| **Mutant** | **Forward primer** | **Reverse primer** |
| --- | --- | --- |
| TM^insWAP^ | GTGGGCATCTTGTGGGCTCCGCTGGTGTTGATGGCTGTG | CATCAACACCAGCGGAGCCCACAAGATGCCCACGATCAG |
| TM^R625C^ | CTGATATATcgctgcAGACTTATGAAGCAAGACTTC | GTCTTGCTTCATAAGTCTgcagcgATATATCAGAGA |
| ΔLVVT | TCTCGGGCACATACTTACCTGGAGCCTG | CTCCAGGTAAGTATGTGCCCGAGAGATCAGGGTTC |
| TM^Δ5^ | GTGTTGATGGCTCTGATAAGGCGCAGAC | CTGCGCCTATATATCAGAGCCATCAACACCAGCAA |
